# Supplementary material for: Selection rules in symmetry-broken systems by symmetries in synthetic dimensions
Source: Nat Commun. 2022 Mar 14;13:1312. doi: 10.1038/s41467-022-29080-3 (PMC8921280; doi:10.1038/s41467-022-29080-3)
Supplement: Supplementary file 1 — Supplementary info [file 41467_2022_29080_MOESM1_ESM.pdf]

Supplementary information:
Selection rules in symmetry-broken systems by symmetries in synthetic dimensions

Matan Even Tzur[1], Ofer Neufeld[1,2], Eliyahu Bordo[1], Avner Fleischer[3], and Oren Cohen[1]

[1] Solid State Institute and Physics Department, Technion-Israel Institute of Technology, Haifa 3200003, Israel

[2] Max Planck Institute for the Structure and Dynamics of Matter and Center for Free-Electron Laser Science, Hamburg, Germany, 22761.

[3] Raymond and Beverly Sackler Faculty of Exact Science, School of Chemistry and Center for Light-Matter-Interaction, Tel Aviv University, Tel-Aviv 6997801, Israel

This supplementary material file contains the derivations of the analytical results presented in Table 2 in the main text, as well as complementary analytical, numerical, and experimental results.

In section I we present the methodology used in the numerical simulations presented in Figure 2 of the main text. In Section II we derive the synthetic dimensions operations $\hat{\zeta}_{\hat{X}}$ and corresponding HHG selection rules, for all dynamical symmetry (DS) operations, within the framework of (2+1)D Floquet group theory[1]. In section III, we provide an explicit derivation of the experimentally observed selection rules in symmetry broken system reported in the main text. In section IV, we show that some selection rules derived in section III are consistent with an emission-channel analysis based on conservation laws[3]. Additionally, we analyze more experimentally observed frequency components using both approaches (real-synthetic symmetries and emission-channels). In section V we depict the full Lissajous curves of the experimentally employed driving fields and derive analytically the ellipticity of the bi-elliptical drivers as a function of the quarter waveplate angle. In section VI, we derive above-threshold ionization selection rules corresponding to real-synthetic symmetries. In section VII, we derive a synthetic symmetry imposed by spin-orbit coupling strengths and derive the corresponding harmonic generation selection rules. In section VIII, we demonstrate that real-synthetic symmetries generalize Floquet-band selection rules that were previously obtained by linear-response theory[4], showing that these selection rules are applicable well beyond the regime of linear response.

I. Numerical solution of the time dependent Schrodinger equation

In this section, we describe the numerical procedure for the calculation of the HHG spectra presented in Figure 2 of the main text. The high harmonic generation (HHG) spectra were obtained by numerically solving the single-electron time dependent Schrodinger equation (TDSE) for an atom irradiated by a laser field, in the length gauge. The TDSE was solved on a cartesian grid of 2 spatial dimensions. The TDSE, given in atomic units within the dipole approximation is:

$$i\frac{\partial}{\partial t}\psi(t,x,y) = \left[-\frac{1}{2}\nabla^2 + V(r) + V_{ab}(r) + \mathbf{r}\cdot\mathbf{E}(r,t)\right]\psi(t,x,y) \quad \text{(I.1)}$$

where $\mathbf{E}(\mathbf{r},t)$ is the laser electric field, and $V(\mathbf{r})$ is the atomic potential, modeled as a softened coulomb potential[5]:

$$V(\mathbf{r}) = -\frac{1}{\sqrt{r^2+a}} \quad \text{(I.2)}$$

The parameter a is set to $a = 0.1195 a.u.$, to match the ionization potential of Ne, that is $I_p = 0.7924\ hartree$ [6]. $V_{ab}(\mathbf{r})$ is a complex absorbing potential, that was included in the equation to avoid nonphysical reflections of the wavefunction from the grid boundaries

$$V_{ab}(\mathbf{r}) = \begin{cases} -i5\times10^{-4}(r-r_0)^3, & r \geq r0 \\ 0, & otherwise \end{cases} \quad \text{(I.3)}$$

where $r_0 = 36\ bohr$. The TDSE was solved by a 3rd order split step method[7,8], starting from the ground state of the model Ne atom. The ground state was found by representing field-free Hamiltonian in matrix form on the cartesian spatial grid, and diagonalizing it. The kinetic energy operator was represented using the finite difference approximation on a two-dimensional grid. In MATLAB, it is obtained by the syntax:

```
I = speye(Nx)
e = ones(Nx,1);
kin = (1/5040)*spdiags([-9*e 128*e -1008*e 8064*e -14350*e 8064*e -1008*e      (I.4)
128*e -9*e],[-4 -3 -2 -1 0 1 2 3 4],Nx,Nx)
Tmat = (-1/2)*(1/(dx^2)).*(kron(kin,I)+kron(I,kin))
```

Here, Nx is the number points on the x-axis and dx is the grid spacing. The command speye assigns a sparse identity matrix to I, the command ones assigns an array whose entries are 1, the variable *kin* is a representation of the operator d^2/dx^2 on a 1D grid, and the variable Tmat is the matrix representing the kinetic energy operator on a two-dimensional grid. *Kron* is the Kronecker tensor multiplication operation. The time dependent propagation employed a trapezoid temporal envelope of the electric field, with 5-cycle long rise and fall sections and a 5-cycle long flat top section. Calculations were carried out on a square cartesian spatial grid, spanning from $x_{min} =$

$y_{min} = -60\ bohr$ to $x_{max} = y_{max} = 60\ bohr$, with a grid spacing of $dx = dy = 0.0586\ bohr$ and a timestep of $dt = 0.011\ a.u.$ The dipole acceleration was calculated by Ehrenfest's theorem:

$$\mathbf{a}(t) = -\langle\psi(\mathbf{r},t)|\boldsymbol{\nabla}V(\mathbf{r}) + \mathbf{E}(t)|\psi(\mathbf{r},t)\rangle \tag{I.5}$$

From which, the harmonic spectra were obtained by a Fourier transform

$$\tilde{\mathbf{E}}(\Omega) = F.T\{\mathbf{a}(t)\} \tag{I.6}$$

II. Derivation of synthetic symmetry operations and corresponding harmonic generation selection rules

In this section, we derive the synthetic dynamical symmetries presented in Table 2 of the main text. Additionally, we derive the corresponding selection rules for high harmonic generation (HHG). We consider a system whose Hamiltonian is given by

$$\hat{H} = \hat{H}_0 + \hat{W} \quad \text{(II.1)}$$

$$\hat{W} = \Re\{\mathbf{Q} \cdot \mathbf{r} e^{is\omega t}\}$$

where $\hat{H}_0(t) = \hat{H}_0(t+T)$ and the Flouqet Hamiltonian $\hat{\mathcal{H}}_f \equiv \hat{H}_0 - i\partial_t$ commutes with the DS operation $\hat{X}$, that is, $[\hat{\mathcal{H}}_f, \hat{X}] = 0$. The operations $\hat{X}$ were systematically tabulated in ref[1], which also provides explicit examples for $\hat{X}$-symmetric Floquet systems for all DSs in $(2+1)D$ and $(3+1)D$. Figure SII.I illustrates the Lissajous curves of these exemplary fields for all (2+1)D Floquet group symmetries.

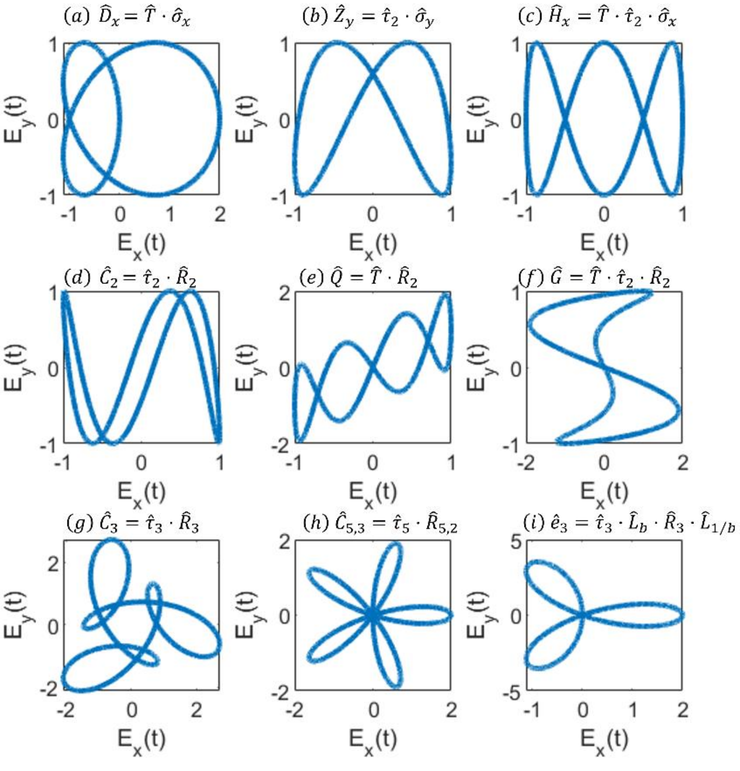

Figure SII.1: Exemplary fields exhibiting Floquet group symmetries in (2+1)D. ***(a)*** *$\hat{D}_x$ symmetry for the example field $\boldsymbol{E}(t) = (cos(\omega t) + cos(2\omega t))\hat{x} + sin(2\omega t)\hat{y}$* ***(b)*** *$\hat{Z}_y$ symmetry for the example field $\boldsymbol{E}(t) = sin(\omega t)\hat{x} + sin(2\omega t + pi/5)$* ***(c)*** *$\hat{H}_x$ symmetry for the example field $\boldsymbol{E}(t) = sin(\omega t)\hat{x} + cos(3\omega t)\hat{y}$* ***(d)*** *$\hat{C}_2$ symmetry for the example field $\boldsymbol{E}(t) = sin(\omega t)\hat{x} + sin\left(3\omega t + \frac{\pi}{7}\right)\hat{y}$* ***(e)*** *$\hat{Q}$ symmetry for the example field $\boldsymbol{E}(t) = sin(\omega t)\hat{x} + (sin(\omega t) + sin(4\omega t))\hat{y}$* ***(f)*** *$\hat{G}$ symmetry for the example field $\boldsymbol{E}(t) = (sin(2\omega t) + cos(3\omega t))\hat{x} + cos(\omega t)\hat{y}$* ***(g)*** *$\hat{C}_3$ symmetry for the example field $\boldsymbol{E}(t) = (cos(\omega t) + cos(2\omega t) + sin\,(4\omega t))\hat{x} + (sin(\omega t) - sin(2\omega t) - cos(4\omega t))\hat{y}$* ***(h)*** *$\hat{C}_{5,3}$ symmetry for the example field $\boldsymbol{E}(t) = (cos(3\omega t) + cos(2\omega t))\hat{x} + (sin(3\omega t) - sin(2\omega t))\hat{y}$.* ***(i)*** *$\hat{e}_3$ symmetry for the example field $\boldsymbol{E}(t) = (cos(\omega t) + cos(2\omega t))\hat{x} + b(sin(\omega t) - sin(2\omega t))\hat{y}$.*

The vector $\mathbf{Q}$ is a complex polarization vector, $T = 2\pi/\omega$, and s is a rational number. The Hamiltonian $\hat{H}$ exhibits a real-synthetic symmetry of the form $\hat{X} \cdot \hat{\zeta}_{\hat{X}}$, where $\hat{\zeta}_{\hat{X}}$ solves the equation

$$\widehat{W} = \Re\left\{\hat{\zeta}_{\hat{X}}(\mathbf{Q}) \cdot \hat{X}\left(\mathbf{r}e^{is\omega t}\right)\right\} \tag{II.2}$$

$\hat{X} \cdot \hat{\zeta}$ imposes the following condition on $\mathbf{E}_{\mathrm{HHG}}(t, \mathbf{Q})$, the emitted field:

$$\mathbf{E}_{\mathrm{HHG}}(\mathrm{t}, \mathbf{Q}) = \hat{\mathrm{X}}\mathbf{E}_{\mathrm{HHG}}\left(t, \hat{\zeta}_{\hat{X}}(\mathbf{Q})\right) \tag{II.3}$$

The amplitude of the nth harmonic, $\mathbf{E}_n(\mathbf{Q})$, is defined by

$$\mathbf{E}_{\mathrm{HHG}}(\mathrm{t}, \mathbf{Q}) = \sum_{\mathrm{n}} \mathbf{E}_n(\mathbf{Q})\mathrm{e}^{\mathrm{i\omega nt}} \tag{II.4}$$

$\hat{X} \cdot \hat{\zeta}$ imposes the selection rules (II.4), which can be reformulated as a selection rule on the expansion coefficient $E_{\mathrm{nx}}^{(\mathrm{abcd})}, E_{\mathrm{ny}}^{(\mathrm{abcd})}$ defined by

$$\mathbf{E}_{\mathrm{n}}(\mathbf{Q}) \equiv \sum_{a,\mathrm{b,c,d}=0}^{\infty} \mathbf{E}_{\mathrm{n}}^{(abcd)} q_{\mathrm{x}}^{\mathrm{a}} q_{\mathrm{y}}^{\mathrm{b}} \bar{\mathrm{q}}_{\mathrm{x}}^{\mathrm{c}} \bar{q}_{\mathrm{y}}^{\mathrm{d}} = \sum_{a,b,\mathrm{c,d}=0}^{\infty} \begin{pmatrix} E_{\mathrm{nx}}^{(\mathrm{abcd})} \\ E_{\mathrm{ny}}^{(\mathrm{abcd})} \end{pmatrix} q_{\mathrm{x}}^{\mathrm{a}} q_{\mathrm{y}}^{\mathrm{b}} \bar{\mathrm{q}}_{\mathrm{x}}^{\mathrm{c}} \bar{q}_{\mathrm{y}}^{\mathrm{d}} \tag{II.5}$$

Time reversal symmetry

Assume that $\hat{H}_0$, the unpertrubed Hamiltonian, exhibits time reversal symmetry (denoted $\hat{T}$ and operates as $t \to -t$), is perturbed by a monochromatic laser electric field of polarization $\mathbf{Q}$ and frequency $s\omega$. Generally, the perturbation $\widehat{W} = (\mathbf{Q} \cdot \mathbf{r})e^{is\omega t} + c.c.$ does not exhibit $\hat{T}$ symmetry since

$$\hat{T}\left[\widehat{W}\right] \equiv \hat{T}^{\dagger}\widehat{W}\hat{T} = (\mathbf{Q} \cdot \mathbf{r})e^{-is\omega t} + c.c. \tag{II.6}$$

However, the perturbed Hamiltonian exhibits the symmetry $\hat{T} \cdot \hat{\zeta}_{\hat{T}}$ where

$$\hat{\zeta}_{\hat{T}}(\mathbf{Q}) = \bar{\mathbf{Q}} \tag{II.7}$$

Explicitly,

$$\hat{T} \cdot \hat{\zeta}_{\hat{T}}\left[\widehat{W}\right] = (\bar{\mathbf{Q}} \cdot \mathbf{r})e^{-is\omega t} + c.c. = \widehat{W} \tag{II.8}$$

The real-synthetic symmetry $\hat{T} \cdot \hat{\zeta}_{\hat{T}}$ results in

$$\hat{T} \cdot \hat{\zeta}_{\hat{T}}\mathbf{E}(\mathrm{t}, \mathbf{Q}) = \sum_{\mathrm{n}} \mathbf{E}_n(\bar{\mathbf{Q}})\mathrm{e}^{-\mathrm{in}\omega \mathrm{t}} = \sum_{\mathrm{n}} \mathbf{E}_n(\mathbf{Q})\mathrm{e}^{\mathrm{in}\omega \mathrm{t}} \tag{II.9}$$

$$\mathbf{E}_n(\bar{\mathbf{Q}}) = \mathbf{E}_{-n}(\mathbf{Q}) = \bar{\mathbf{E}}_n(\mathbf{Q}) \tag{II.10}$$

By Eq.(II.5), we obtain the selection rule

$$\mathbf{E}_{\mathrm{n}}^{(abcd)} \in \mathbb{R} \tag{II.11}$$

$\hat{Q} = \hat{T} \cdot \hat{R}_2$ symmetry

Assume that $\hat{H}_0$, the unpertrubed Hamiltonian, exhibits $\hat{Q} = \hat{T} \cdot \hat{R}_2$ symmetry, where $\hat{R}_2$ is a π rotation. The perturbation $\hat{W}$ does not exhibit $\hat{Q}$ symmetry since

$$\hat{Q}[\hat{W}] = -(\mathbf{Q} \cdot \mathbf{r})e^{-is\omega t} + c.c. \tag{II.12}$$

Note that $\hat{Q}$ is the symmetry operation while $\mathbf{Q}$ is the polarization of the symmetry breaking field. The perturbed Hamiltonian exhibits the symmetry $\hat{Q} \cdot \hat{\zeta}_{\hat{Q}}$ where

$$\hat{\zeta}_{\hat{Q}}(\mathbf{Q}) = -\bar{\mathbf{Q}} \tag{II.13}$$

since

$$\hat{Q} \cdot \hat{\zeta}_{\hat{Q}}[\hat{W}] = (-\bar{\mathbf{Q}}) \cdot (-\mathbf{r})e^{-is\omega t} + c.c. = \hat{W} \tag{II.14}$$

$\hat{\mathrm{Q}} \cdot \hat{\zeta}_{\hat{Q}}$ results in the selection rule

$$\hat{Q} \cdot \hat{\zeta}_{\hat{Q}} \mathbf{E}_{\mathrm{HHG}}(t, \mathbf{Q}) = \sum_n -\mathbf{E}_n(-\bar{\mathbf{Q}})e^{-in\omega t} = \sum_n \mathbf{E}_n(\mathbf{Q})e^{in\omega t} \tag{II.15}$$

$$\bar{\mathbf{E}}_n(\mathbf{Q}) = -\mathbf{E}_n(-\bar{\mathbf{Q}}) \tag{II.16}$$

By Eq.(II.5), we obtain the selection rule

$$\begin{pmatrix} E_{nx}^{(abcd)} \\ E_{ny}^{(abcd)} \end{pmatrix} \in i^{1+a+b+c+d}\mathbb{R} \tag{II.17}$$

$\hat{G} = \hat{T} \cdot \hat{\boldsymbol{\tau}}_2 \cdot \hat{R}_2$ symmetry

The perturbation $\hat{W}$ does not exhibit $\hat{G}$ symmetry since

$$\hat{G}[\hat{W}] = (\mathbf{Q}) \cdot ((-1)^{s+1}\mathbf{r})e^{-is\omega t} + c.c. \tag{II.18}$$

The perturbed Hamiltonian exhibits the symmetry $\hat{G} \cdot \hat{\zeta}_{\hat{G}}$ where

$$\hat{\zeta}_{\hat{G}}(\mathbf{Q}) = (-1)^{s+1}\bar{\mathbf{Q}} \tag{II.19}$$

since

$$\hat{G} \cdot \hat{\zeta}_{\hat{G}}[\hat{W}] = ((-1)^{s+1}\bar{\mathbf{Q}}) \cdot ((-1)^{s+1}\mathbf{r})e^{-is\omega t} + c.c. = \hat{W} \tag{II.20}$$

$\hat{G} \cdot \hat{\zeta}_{\hat{G}}$ results in

$$\widehat{\mathrm{G}}\cdot\hat{\zeta}_{\hat{G}}\mathbf{E}_{\mathrm{HHG}}(t,\mathbf{Q})=\sum_{\mathrm{n}}(-1)^{\mathrm{n}+1}\mathbf{E}_{n}\left((-1)^{(s+1)}\overline{\mathbf{Q}}\right)\mathrm{e}^{-\mathrm{in}\omega\mathrm{t}}=\sum_{\mathrm{n}}\mathbf{E}_{n}(\mathbf{Q})\mathrm{e}^{\mathrm{in}\omega\mathrm{t}} \tag{II.21}$$

$$\overline{\mathbf{E}}_{n}(\mathbf{Q})=(-1)^{\mathrm{n}+1}\mathbf{E}_{n}\left((-1)^{(s+1)}\overline{\mathbf{Q}}\right) \tag{II.22}$$

By Eq.(II.5), we obtain

$$\overline{\begin{pmatrix}E_{nx}^{(abcd)}\\E_{ny}^{(abcd)}\end{pmatrix}}=(-1)^{n+1+(s+1)(a+b+c+d)}\begin{pmatrix}E_{nx}^{(a+b+c+d)}\\E_{ny}^{(a+b+c+d)}\end{pmatrix} \tag{II.23}$$

hence

$$\begin{pmatrix}E_{nx}^{(abcd)}\\E_{ny}^{(abcd)}\end{pmatrix}\in i^{n+1+(s+1)(a+b+c+d)}\mathbb{R}^{2} \tag{II.24}$$

$\hat{\boldsymbol{C}}_{\mathbf{2}}=\hat{\boldsymbol{\tau}}_{\mathbf{2}}\cdot\hat{\boldsymbol{R}}_{\mathbf{2}}$ symmetry

Assume that $\widehat{H}_0$, the unperturbed Hamiltonian, exhibits $\hat{C}=\hat{\tau}_2\cdot\hat{R}_2$ symmetry. The perturbation $\widehat{W}$ does not exhibit $\hat{C}_2$ symmetry since

$$\hat{C}_2\left[\widehat{W}\right]=(-1)^{s+1}(\mathbf{Q}\cdot\mathbf{r})e^{is\omega t}+c.c. \tag{II.25}$$

The perturbed Hamiltonian exhibits the symmetry $\hat{C}_2\cdot\hat{\zeta}_{\hat{C}_2}$ where

$$\hat{\zeta}_{\hat{C}_2}(\mathbf{Q})=(-1)^{s+1}\mathbf{Q} \tag{II.26}$$

since

$$\hat{C}_2\cdot\hat{\zeta}_{\hat{C}_2}\left[\widehat{W}\right]=((-1)^{s+1}\mathbf{Q})\cdot((-1)^{s+1}\mathbf{r})e^{is\omega t}+c.c.=\widehat{W} \tag{II.27}$$

By Eq.(II.5), we obtain the selection rule

$$\begin{pmatrix}E_{nx}^{(abcd)}\\E_{ny}^{(abcd)}\end{pmatrix}=(-1)^{n+1+(s+1)(a+b+c+d)}\begin{pmatrix}E_{nx}^{(abcd)}\\E_{ny}^{(abcd)}\end{pmatrix} \tag{II.28}$$

Therefore,

$$n+(s+1)(a+b+c+d)=2q\Rightarrow\mathbf{E}_{n}^{(a+b+c+d)}=\mathbf{0} \tag{II.29}$$

$\hat{\boldsymbol{Z}}_{\boldsymbol{y}}=\hat{\boldsymbol{\tau}}_{\mathbf{2}}\cdot\hat{\boldsymbol{\sigma}}_{\boldsymbol{y}}$ symmetry

Assume that $\widehat{H}_0$ exhibits $\hat{Z}_y=\hat{\tau}_2\cdot\hat{\sigma}_y$ symmetry. The perturbation $\widehat{W}$ does not exhibit $\hat{Z}_y$ symmetry since

$$\hat{Z}_y\left[\widehat{W}\right]=\mathbf{Q}\cdot\left(\begin{pmatrix}(-1)^{s+1}&0\\0&(-1)^{s}\end{pmatrix}\mathbf{r}\right)e^{is\omega t}+c.c. \tag{II.30}$$

The perturbed Hamiltonian exhibits the symmetry $\hat{Z}_y \cdot \hat{\zeta}_{\hat{Z}_y}$ where

$$\hat{\zeta}_{\hat{Z}_y}(\mathbf{Q}) = \begin{pmatrix} (-1)^{s+1} & 0 \\ 0 & (-1)^s \end{pmatrix} \begin{pmatrix} q_x \\ q_y \end{pmatrix} \tag{II.31}$$

$\hat{Z}_y \cdot \hat{\zeta}_{\hat{Z}_y}$ results in

$$\begin{aligned} \hat{Z}_y \cdot \hat{\zeta}_{\hat{Z}_y} \mathbf{E}_{\mathrm{HHG}}(t, \mathbf{Q}) &= \sum_n \hat{\sigma}_y (-1)^n \mathbf{E}_n \left(\hat{\zeta}_{\hat{Z}_y}(\mathbf{Q}) \right) e^{in\omega t} \\ &= \sum_n \mathbf{E}_n(\mathbf{Q}) e^{in\omega t} \end{aligned} \tag{II.32}$$

By Eq.(II.5),

$$\begin{aligned} (-1)^{n+1+(s+1)(a+c)+s(b+d)} E_{nx}^{(abcd)} &= E_{nx}^{(abcd)} \\ (-1)^{n+(s+1)(a+c)+s(b+d)} E_{ny}^{(abcd)} &= E_{ny}^{(abcd)} \end{aligned} \tag{II.33}$$

Hence $\hat{Z}_y \cdot \hat{\zeta}_{\hat{Z}_y}$ results in the following selection rules

$$n + (s+1)(a+c) + s(b+d) = 2q \Rightarrow E_{nx}^{(abcd)} = 0 \tag{II.34}$$

$$n + (s+1)(a+c) + s(b+d) = 2q + 1 \Rightarrow E_{ny}^{(abcd)} = 0 \tag{II.35}$$

$\widehat{\boldsymbol{D}}_{\boldsymbol{y}} = \widehat{\boldsymbol{T}} \cdot \widehat{\boldsymbol{\sigma}}_{\boldsymbol{y}}$ symmetry

Assume that $\widehat{H}_0$ exhibits $\widehat{D} = \widehat{T} \cdot \hat{\sigma}_y$ symmetry. The perturbation $\widehat{W}$ does not exhibit $\widehat{D}_y$ symmetry since

$$\widehat{D}_y[\widehat{W}] = \left(\mathbf{Q} \cdot \left(\begin{pmatrix} -1 & 0 \\ 0 & 1 \end{pmatrix} \mathbf{r} \right) \right) e^{-is\omega t} + c.c. \tag{II.36}$$

The perturbed Hamiltonian exhibits the symmetry $\widehat{D}_y \cdot \hat{\zeta}_{\widehat{D}_y}$ where

$$\hat{\zeta}_{\widehat{D}_y}(\mathbf{Q}) = \begin{pmatrix} -1 & 0 \\ 0 & 1 \end{pmatrix} \overline{\mathbf{Q}} \tag{II.37}$$

since

$$\widehat{D}_y \cdot \hat{\zeta}_{\widehat{D}_y}[\widehat{W}] = \left(\begin{pmatrix} -1 & 0 \\ 0 & 1 \end{pmatrix} \overline{\mathbf{Q}} \cdot \begin{pmatrix} -1 & 0 \\ 0 & 1 \end{pmatrix} \mathbf{r} \right) e^{-is\omega t} + c.c. = \widehat{W} \tag{II.38}$$

Operating with $\widehat{D}_y \cdot \hat{\zeta}_{\widehat{D}_y}$ on Eq.(II.4), we obtain

$$\widehat{D}_y \cdot \hat{\zeta}_{\widehat{D}_y} \mathbf{E}_{\mathrm{HHG}}(t, \mathbf{Q}) = \sum_n \hat{\sigma}_y \mathbf{E}_n \left(\begin{pmatrix} -1 & 0 \\ 0 & 1 \end{pmatrix} \overline{\mathbf{Q}} \right) e^{-in\omega t} = \sum_n \mathbf{E}_n(\mathbf{Q}) e^{in\omega t} \tag{II.39}$$

$$(-1)^{1+a+c}E_{nx}^{(abcd)} = \bar{E}_{nx}^{(abcd)}$$

$$(-1)^{a+c}E_{ny}^{(abcd)} = \bar{E}_{ny}^{(abcd)}$$

By Eq.(II.5)

$$E_{nx}^{(abcd)} \in i^{1+a+c}\mathbb{R} \tag{II.40}$$

$$E_{ny}^{(abcd)} \in i^{a+c}\mathbb{R} \tag{II.41}$$

$\hat{\boldsymbol{H}}_{\boldsymbol{y}} = \hat{\boldsymbol{\tau}}_{\boldsymbol{2}} \cdot \hat{\boldsymbol{T}} \cdot \hat{\boldsymbol{\sigma}}_{\boldsymbol{y}}$ symmetry

Assume that $\hat{H}_0$ exhibits $\hat{H}_y = \hat{\tau}_2 \cdot \hat{T} \cdot \hat{\sigma}_y$ symmetry. The perturbation $\hat{W}$ does not exhibit $\hat{H}_y$ symmetry since

$$\hat{H}_y\left[\hat{W}\right] = \left(\mathbf{Q} \cdot \left(\begin{pmatrix} (-1)^{s+1} & 0 \\ 0 & (-1)^{s} \end{pmatrix} \mathbf{r}\right)\right) e^{-is\omega t} + c.c. \tag{II.42}$$

The perturbed Hamiltonian exhibits the symmetry $\hat{H}_y \cdot \hat{\zeta}_{\hat{H}_y}$ where

$$\hat{\zeta}_{\hat{H}_y}(\mathbf{Q}) = \begin{pmatrix} (-1)^{s+1} & 0 \\ 0 & (-1)^{s} \end{pmatrix} \bar{\mathbf{Q}} \tag{II.43}$$

since

$$\hat{H}_y \cdot \hat{\zeta}_{\hat{H}_y}\left[\hat{W}\right] = \left(\begin{pmatrix} (-1)^{s+1} & 0 \\ 0 & (-1)^{s} \end{pmatrix} \bar{\mathbf{Q}} \cdot \begin{pmatrix} (-1)^{s+1} & 0 \\ 0 & (-1)^{s} \end{pmatrix} \mathbf{r}\right) e^{-is\omega t} + c.c. \\ = \hat{W} \tag{II.44}$$

Operating with $\hat{H}_y \cdot \hat{\zeta}_{\hat{H}_y}$ on Eq.(II.4) we obtain

$$\hat{H}_y \cdot \hat{\zeta}_{\hat{H}_y} \mathbf{E}_{\mathrm{HHG}}(t, \boldsymbol{Q}) = \sum_{\mathrm{n}} \begin{pmatrix} (-1)^{n+1} & 0 \\ 0 & (-1)^{n} \end{pmatrix} \mathbf{E}_n \left(\begin{pmatrix} (-1)^{s+1} & 0 \\ 0 & (-1)^{s} \end{pmatrix} \bar{\mathbf{Q}}\right) \mathrm{e}^{-\mathrm{in}\omega \mathrm{t}} \\ = \sum_{\mathrm{n}} \mathbf{E}_n(\mathbf{Q}) \mathrm{e}^{\mathrm{in}\omega \mathrm{t}} \tag{II.45}$$

By Eq.(II.5)

$$E_{nx}^{(abcd)} \in i^{n+1+(s+1)(\mathrm{a}+\mathrm{c})+s(\mathrm{b}+\mathrm{d})}\mathbb{R} \tag{II.46}$$

$$E_{ny}^{(abcd)} \in i^{n+(s+1)(a+c)+s(\mathrm{b}+\mathrm{d})}\mathbb{R} \tag{II.47}$$

$\hat{\boldsymbol{C}}_{\boldsymbol{NM}} = \hat{\boldsymbol{R}}_{\boldsymbol{N,M}} \cdot \hat{\boldsymbol{\tau}}_{\boldsymbol{N}}$ symmetry

Next, we consider Floquet systems that exhibit higher order rotational DSs that are similarly perturbed by a monochromatic laser beam. The high order rotational DS are defined as $\hat{C}_{N,M} =$

$\hat{\tau}_N \cdot \hat{R}_{N,M}$ where $\hat{\tau}_N$ is a T/N time translation and $\hat{R}_{N,M}$ is a $2\pi M/N$ rotation. Upon an external perturbation whose polarization vector is $\mathbf{Q}$ with frequency $s\omega$, the $\hat{C}_{NM}$ DS breaks, and reduces to the synthetic symmetry $\hat{C}_{N,M} \cdot \hat{\zeta}_{\hat{C}_{NM}}$ where

$$\hat{\zeta}_{\hat{C}_{NM}}(\mathbf{Q}) = e^{-\frac{i2\pi s}{N}} \hat{R}^{(\mathbf{Q})}{}_{N,M} \cdot \mathbf{Q} \tag{II.48}$$

where $\hat{R}^{(\mathbf{Q})}{}_{N,M}$ is a $2\pi M/N$ rotation in the two-dimensional synthetic $\mathbf{Q}$ space. This can be seen by considering how the perturbation term $\widehat{W} = \mathbf{Q} \cdot \mathbf{r} e^{is\omega t} + c.c.$ transforms under the operation $\hat{C}_{N,M} \cdot \hat{\zeta}_{\hat{C}_{NM}}$:

$$\hat{C}_{N,M} \cdot \hat{\zeta}_{\hat{C}_{NM}}[\widehat{W}] = \left(e^{-\frac{i2\pi s}{N}} \hat{R}^{(\mathbf{Q})}_{N,M}\mathbf{Q}\right) \cdot \left(\hat{R}_{N,M}\mathbf{r}\right) e^{\frac{i2\pi s}{N}} e^{is\omega t} + c.c. = \widehat{W} \tag{II.49}$$

To derive the harmonic generation selection rules for this case we first write the amplitude of the nth harmonic $\mathbf{E}_n(\mathbf{Q})$ in as a sum of right/left circularly polarized (RCP/LCP) vectors, and expand it in terms of the right/left-handed circularly polarized components of the perturbation:

$$\begin{aligned} \mathbf{E}_n(\mathbf{Q}) &= E_{Rn}(\mathbf{Q})(\hat{x} - i\hat{y}) + E_{Ln}(\mathbf{Q})(\hat{x} + i\hat{y}) \\ &= \sum_{k,l,h,j=0}^{\infty} \Big(E_{Rn}^{(abcd)}(\hat{x} - i\hat{y}) \\ &\quad + E_{Ln}^{(abcd)}(\hat{x} + i\hat{y})) \Big) q_R^a q_L^b \bar{q}_R^c \bar{q}_L^d \end{aligned} \tag{II.50}$$

where $\hat{x}, \hat{y}$ are Cartesian basis vectors, $q_{R,L} = q_x \mp i q_y$ are the RCP and LCP of $\mathbf{Q}$, $E_{Rn} = E_{nx} \mp iE_{ny}$, and $E_{Rn}^{(klhj)}, E_{Ln}^{(klhj)}$ are the coefficients of the polynomial expansion. Again, we utilize the invariance of the time-dependent emission under the symmetry operation and write $\hat{C}_{N,M} \cdot \hat{\zeta}_{\hat{C}_{NM}} \mathbf{E}_{\text{HHG}}(t, \mathbf{Q}) = \mathbf{E}_{\text{HHG}}(t, \mathbf{Q})$, that is

$$\hat{R}_{N,M}\mathbf{E}_{\text{HHG}}\left(t + T/N\,, e^{-\frac{i2\pi s}{N}} \hat{R}^{(\mathbf{Q})}{}_{N,M} \cdot \mathbf{Q}\right) = \mathbf{E}_{\text{HHG}}(t, \boldsymbol{Q}) \tag{II.51}$$

which translate to the following equation for the amplitude of the *n'th* harmonic, $\mathbf{E}_n(\mathbf{Q})$*:*

$$e^{\frac{2\pi i n}{N}} \hat{R}_{N,M}\mathbf{E}_n\left(e^{-\frac{i2\pi s}{N}} \hat{R}^{(\boldsymbol{Q})}{}_{N,M} \cdot \mathbf{Q}\right) = \mathbf{E}_n(\mathbf{Q}) \tag{II.52}$$

The RCP and LCP unit vector $(\hat{x} \mp i\hat{y})$ are eigenvectors of $\hat{R}_{N,M}$ with eigenvalues $e^{-i2\pi M/N}, e^{i2\pi M/N}$ respectively. Hence,

$$e^{\frac{2\pi i}{N}[n - M(a-b-c+d) - s(a+b-c-d) - M]} E_{Rn}^{(abcd)} = E_{Rn}^{(abcd)} \tag{II.53}$$

$$e^{\frac{2\pi i}{N}[n - M(a-b-c+d) - s(a+b-c-d)) + M]} E_{Ln}^{(abcd)} = E_{Ln}^{(abcd)} \tag{II.54}$$

Hence, there is a $q_R^a q_L^b \bar{q}_R^c \bar{q}_L^d$ contribution to $E_{Rn}(\mathbf{Q})$ *(*$E_{Ln}(\mathbf{Q})$) only if

$$(n - M(\mathrm{a-b-c+d}) - s(\mathrm{a+b-c-d}) \mp M) = 0\ (mod\ N) \qquad \text{(II.55)}$$

where $mod\ N$ is the modulo operation of basis N.

$\hat{\boldsymbol{e}}_{NM} = \left(\hat{\boldsymbol{L}}_{\boldsymbol{b}} \cdot \hat{\boldsymbol{R}}_{N,M} \cdot \hat{\boldsymbol{L}}_{\boldsymbol{1/b}}\right) \cdot \hat{\boldsymbol{\tau}}_{N}$ symmetry

Next, we consider Floquet systems that exhibit high order elliptical DSs that are similarly perturbed by a monochromatic laser beam. The high order elliptical DS of ellipticity b is defined as $\hat{e}_{NM} = \left(\hat{L}_b \cdot \hat{R}_{N,M} \cdot \hat{L}_{1/b}\right) \cdot \hat{\tau}_N$ where $\hat{\tau}_N$ is a T/N time translation, $\hat{R}_{N,M}$ is a $2\pi M/N$ rotation, and $\hat{L}_b$ is expressed in the cartesian basis as

$$\hat{L}_b = \begin{pmatrix} 1 & 0 \\ 0 & b \end{pmatrix} \qquad \text{(II.56)}$$

Upon an external perturbation whose polarization vector is $\mathbf{Q}$ and with frequency $s\omega$, the $\hat{e}_{NM}$ DS breaks, and reduces to the symmetry $\hat{e}_{N,M} \cdot \hat{\zeta}_{\hat{e}_{NM}}$ where

$$\hat{\zeta}_{\hat{e}_{NM}}(\mathbf{Q}) = e^{-\frac{i2\pi s}{N}} \hat{L}^{(\mathbf{Q})}{}_{1/b} \cdot \hat{R}^{(\mathbf{Q})}{}_{N,M} \cdot \hat{L}^{(\mathbf{Q})}{}_{b} \cdot \mathbf{Q} \qquad \text{(II.57)}$$

Here, $\hat{L}^{(\mathbf{Q})}{}_{1/b} \cdot \hat{R}^{(\mathbf{Q})}{}_{N,M} \cdot \hat{L}^{(\boldsymbol{Q})}{}_{b}$ is a generalized elliptical rotation of ellipticity $1/_b$ in the two-dimensional synthetic $\mathbf{Q}$ space.

To derive the harmonic generation selection rules for this case we first write the amplitude of the $\mathrm{n^{th}}$ harmonic $\mathbf{E}_\mathrm{n}(\mathbf{Q})$ as a sum of right/left elliptically polarized (REP/LEP) vectors, and expand it in terms of the right/left elliptically polarized components of the perturbation:

$$\begin{aligned} \mathbf{E}_\mathrm{n}(\mathbf{Q}) &= E_{-n}(\mathbf{Q})(\hat{x} - ib\hat{y}) + E_{+n}(\mathbf{Q})(\hat{x} + ib\hat{y}) \\ &= \sum_{a,\mathrm{b,c,d}=0}^{\infty} \left(E_{-\mathrm{n}}^{(abcd)}(\hat{x} - ib\hat{y}) + E_{+\mathrm{n}}^{(abcd)}(\hat{x} \right. \\ &\quad \left. + ib\hat{y})\right) q_-^a q_+^b \bar{q}_-^c \bar{q}_+^d \end{aligned} \qquad \text{(II.58)}$$

where $\hat{x}, \hat{y}$ are Cartesian basis vectors, $q_{-,+} = q_x \mp ibq_y$, $E_{-,+\mathrm{n}} = E_{nx} \mp ibE_{ny}$ are the REP and LEP components of the vectors $\mathbf{Q}$ and $\mathbf{E}_\mathrm{n}(\mathbf{Q})$, and $E_{-n}^{(abcd)}, E_{+n}^{(abcd)}$ are the coefficients of the polynomial expansion. Again, we utilize the invariance of the time-dependent emission under the symmetry operation and write $\hat{e}_{N,M} \cdot \hat{\zeta}_{\hat{e}_{N,M}} \mathbf{E}_\mathrm{HHG}(t, \mathbf{Q}) = \mathbf{E}_{HHG}(t, \mathbf{Q})$, that is

$$(\hat{L}_b \cdot \hat{R}_{N,M} \cdot \hat{L}_{1/b})\mathbf{E}\left(t + T/N, e^{-\frac{i2\pi s}{N}} \hat{L}^{(\mathbf{Q})}{}_{1/b} \cdot \hat{R}^{(\boldsymbol{Q})}{}_{N,M} \cdot \hat{L}^{(\mathbf{Q})}{}_{b} \cdot \mathbf{Q}\right) = \mathbf{E}(t, \mathbf{Q}) \qquad \text{(II.59)}$$

which translate to the following equation for the amplitude of the n'th harmonic, $\mathbf{E}_n(\mathbf{Q})$:

$$e^{\frac{2\pi i n}{N}} \hat{L}_b \cdot \hat{R}_{N,M} \cdot \hat{L}_{1/b} \mathbf{E}_n \left(e^{-\frac{i2\pi s}{N}} \hat{L}^{(\boldsymbol{Q})}{}_{1/b} \cdot \hat{R}^{(Q)}{}_{N,M} \cdot \hat{L}^{(\boldsymbol{Q})}{}_{b} \cdot \mathbf{Q}\right) = \mathbf{E}_n(\mathbf{Q}) \qquad \text{(II.60)}$$

The REP and LEP elliptical vectors are eigenvectors of $\hat{e}_{N,M}$ with eigenvalues $e^{-i2\pi M/N}, e^{i2\pi M/N}$ respectively. Hence, plugging Eq.(II.58) into Eq. (II.60), we obtain the following eigenvalue equations:

$$e^{\frac{2\pi i}{N}[n-M(a-b-c+d)-s(a+b-c-d)-M]}E_{-n}^{(abcd)} = E_{-n}^{(abcd)} \qquad \text{(II.61)}$$

$$e^{\frac{2\pi i}{N}[n-M(a-b-c+d)-s(a+b-c-d)+M]}E_{+n}^{(abcd)} = E_{+n}^{(abcd)} \qquad \text{(II.62)}$$

Hence, there is a $\mathrm{q}_-^a \mathrm{q}_+^b \bar{\mathrm{q}}_-^c \bar{\mathrm{q}}_+^d$ contribution to $E_{-\mathrm{n}}(\mathbf{Q})$ ($E_{+\mathrm{n}}(\mathbf{Q})$) only if

$$(n - M(a-b-c+d) - s(a+b-c-d) \mp M) = 0 \ (mod\ N) \qquad \text{(II.63)}$$

where $mod\ N$ is the modulo operation of basis N.

III. Derivation of the experimentally observed selection rules in a symmetry broken system

In this section, we derive explicitly the selection rules observed in the experiment, corresponding to the broken rotational symmetry (i.e., around $\theta = 45°$). We obtained in the main text that

$$\mathbf{E}_{\mathrm{HHG}}(t, \delta_1, \delta_2) = \hat{R}_{2.95}\mathbf{E}_{\mathrm{HHG}}(t + T_{\omega_0}/2.95\,, \delta_1 e^{0.95\times 2\pi i/2.95}, \delta_2 e^{-0.95\times 2\pi i/2.95}) \qquad \text{(III.1)}$$

By expanding $\mathbf{E}_{\mathrm{HHG}}(t, \delta_1, \delta_2)$ to a power series in $\delta_1^a \delta_2^b \overline{\delta_1}^c \overline{\delta_2}^d$

$$\mathbf{E}_{\mathrm{HHG}}(t, \delta_1, \delta_2) = \sum_n \boldsymbol{E}_n^{(abcd)} e^{in\omega_0 t} \delta_1^a \delta_2^b \overline{\delta_1}^c \overline{\delta_2}^d \qquad \text{(III.2)}$$

We obtain

$$\hat{R}_{2.95}\mathbf{E}_{\mathrm{HHG}}\left(t + T_{\omega_0}/2.95\,, \delta_1 e^{0.95\times 2\pi i/2.95}, \delta_2 e^{-0.95\times 2\pi i/2.95}\right) \qquad \text{(III.3)}$$
$$= \sum_n e^{0.95\times 2\pi i\,(a-b-c+d)/2.95} \hat{R}_{2.95} \mathbf{E}_{\mathrm{n}}^{(abcd)} e^{2\pi i n/2.95} e^{in\omega_0 t} \delta_1^a \delta_2^b\, \overline{\delta_1}^c \overline{\delta_2}^d$$

The action of $\hat{R}_{2.95}$ on the vector $\mathbf{E}_n^{(abcd)}$ can be obtained by representing it as a sum circularly polarized vectors, $\mathbf{E}_n^{(abcd)} = E_{Rn}^{(abcd)}(\hat{x} - i\hat{y}) + E_{Ln}^{(abcd)}(\hat{x} + i\hat{y})$ where $\hat{x}, \hat{y}$ are Cartesian basis vectors**.** The vectors $\hat{x} \mp i\hat{y}$ are eigenvectors of the rotation operator $\hat{R}_{2.95}$ that exhibit $\hat{R}_{2.95}(\hat{x} \mp i\hat{y}) = e^{\mp 2\pi i/2.95}(\hat{x} \mp i\hat{y})$**.** Hence, the right-handed $(\hat{x} - i\hat{y})$ and left-handed $(\hat{x} + i\hat{y})$ polarization components of harmonic n satisfy:

$$\mathbf{E}_{R/L\,n}^{(abcd)} = e^{2\pi i\times[n+0.95\times\,(a-b-c+d)\mp 1]/2.95} \mathbf{E}_{R/L\,n}^{(abcd)} \qquad \text{(III.4)}$$

This translates to the general selection rule:

$$(n + 0.95 \times\, (a - b - c + d) \mp 1) = 0\ (mod\ 2.95) = 2.95q \qquad \text{(III.5)}$$

where q is an integer. Firstly, we plug in $n = 20.7 = 2.95 \times 6 + 3$, and $q = 7$

$$3 + 0.95 \times\, (a - b - c + d) \mp 1 = 2.95 \qquad \text{(III.6)}$$

which has a solution for $a - b - c + d = 1$ hence the lowest order allowed contribution is linear in $\delta_{1,2}$, i.e., it is linear in the deviation angle $|\theta - 45°|$.

Next, we plug in $n = 19.75 = 5 \times 2.95 + 5$,

$$5 + 0.95 \times\, (a - b - c + d) \mp 1 = 2.95q \qquad \text{(III.7)}$$

There is no solution for $a - b - c + d = \pm 1$, hence there is no contribution linear in $\delta_{1,2}$. However, there is a quadratic contribution because $(a - b - c + d) = 2$ solves

$$5 + 0.95 \times\, (a - b - c + d) - 1 = 2.95 \times 2 = 5.9 \qquad \text{(III.8)}$$

Finally, let us plug in $n = 18.8 = 4 \times 2.95 + 7$.

$$7 + 0.95 \times (a - b - c + d) \mp 1 = 2.95q \tag{III.9}$$

There is no solution for $a - b - c + d = 1$ because there is no integer q that fulfills

$$7 + 0.95 \mp 1 = 2.95q \tag{III.10}$$

Likewise, there is no solution for $a - b - c + d = 2$, because there is no integer q that fulfills:

$$7 + 0.95 \times 2 \mp 1 = 2.95q \tag{III.11}$$

However, there is a solution for $a - b - c + d = 3$,

$$7 + 0.95 \times 3 \mp 1 = 2.95q = \{q = 3\} = 8.85 \tag{III.12}$$

This is consistent with the observed cubic scaling, i.e., $a = 3$ and $b = c = d = 0$.

This selection rule can also be obtained using the general selection rules in Table 2 of the main text. The selection rule for broken $\hat{C}_{N,M}$ symmetry is

$$n - M(\mathrm{a - b - c + d}) - s(\mathrm{a + b - c - d}) \mp M = 0 \ (\mathrm{mod\ N}) = Nz \tag{III.13}$$

where z is an integer. For the symmetry $\hat{C}_{2.95}$ perturbed by fields with frequencies $s_1 = 1$ and $s_2 = 1.95$, we plug in N=2.95, M=1. We also plug in $s = s_1 = 1$ because s_1 and s_2 result in identical selection rules (through the freedom in the choice of z). The selection rule is reduced to:

$$\exists z \in \mathbb{Z}: n + 2(-\mathrm{a} + \mathrm{c}) \mp 1 = 2.95z \tag{III.14}$$

If $n = 20.7 = 6 \times 2.95 + 3$, we have

$$3 + 2(-\mathrm{a} + \mathrm{c}) \mp 1 = 2.95\mathrm{z} \tag{III.15}$$

Hence the lowest order contribution is a=1, c=0, z=0 (i.e., it is linear).

If $n = 19.75 = 5 \times 2.95 + 5$ we have

$$5 + 2(-\mathrm{a} + \mathrm{c}) \mp 1 = 2.95\mathrm{z} \tag{III.16}$$

Hence the lowest order contribution is a=2, c=0, z=0 (i.e., it is quadratic).

If $n = 18.8 = 2.95 \times 4 + 7$, we have

$$7 + 2(-\mathrm{a} + \mathrm{c}) \mp 1 = 2.95\mathrm{z} \tag{III.17}$$

Hence, the lowest order contribution is a=3, c=0. Similarly, a quartic contribution is also allowed (a=4, c=0).

IV. Consistency of real-synthetic symmetry selection rules with emission-channel analysis based on conservation laws

In this section, we show that some of the selection rules we derived in section III and explored experimentally (see main text), are consistent with an emission channel analysis based on conservation laws[3]. Additionally, we analyze experimentally observed spectral components that were not presented in the main text using both approaches of real-synthetic symmetries and emission-channels.

Emission channel analysis of harmonic orders $\mathbf{18.8}$, $\mathbf{19.75}$ and $\mathbf{20.7}$ $\boldsymbol{at}$ $\theta \sim 45°$.

Firstly, we consider the emission at $\theta = 45°$ exactly, which is governed by standard dynamical symmetry selection rules. At this point, the driving field consists of ω_0-photons with a $\sigma_1 = -1$ spin (corresponding to the RCP polarization) and $1.95\omega_0$ photons with $\sigma_2 = 1$ spin (corresponding to the LCP polarization). Since HHG is a parametric process, the emitted frequencies can are given by

$$\Omega_{(n_1,n_2)} = n_1\omega_0 + 1.95n_2\omega_0 \tag{IV.1}$$

Here, n_1 (n_2) is the number of ω_0 ($1.95\omega_0$) photons annihilated in the generation process of one $\Omega_{(n_1,n_2)}$ photon, where (n_1, n_2) is referred to as an "emission-channel". The spin of the emitted $\Omega_{(n_1,n_2)}$ photon is given by:

$$\sigma_{(n_1,n_2)} = \sigma_1 n_1 + \sigma_2 n_2 = -n_1 + n_2 \tag{IV.2}$$

Here, σ_1 and σ_2 are the spin expectation values of the pump ω_0 photons and $1.95\omega_0$ photons, respectively. Because $\sigma_{(n_1,n_2)}$ represents the spin of an emitted photon, only emission channels that result in $\sigma_{(n_1,n_2)} = \pm 1$ are allowed[3]. Additionally, parity conservation forbids emission channels with an even value of $n_1 + n_2$.

At $\theta = 45°$ we have $\sigma_1 = -1$ and $\sigma_2 = 1$, thus conservation of spin, parity, and energy forbids the generation of harmonic orders 18.8, 19.75, and 20.7, as these harmonic orders correspond to emission channels that exhibit $|\sigma_{(n_1,n_2)}| > 1$ (Table IV.I)

Table IV.I: Harmonic orders 18.8, 19.75, 20.7 are forbidden at $\theta = 45°$ due to conservations of energy, spin and parity.

Harmonic order	n_1	n_2	$n_1 + n_2$	$\sigma_{(n_1,n_2)}$	Allowed/forbidden at $\theta = 45°$
18.8	11	4	15	-7	Forbidden
19.75	10	5	15	-5	Forbidden
20.7	9	6	15	-3	Forbidden

Next, we consider the situation where the QWP angle detunes from 45° and symmetry breaking polarization components are added to the system, corresponding to perturbation photons with frequencies ω_0 and $1.95\omega_0$. In this case, the emission channels are denoted by (n_1, n_2, n_3, n_4) represents the annihilation of n_1 RCP ω_0 photons (pump), n_2 LCP $1.95\omega_0$ photons (pump), n_3

LCP ω_0 photons (perturbation), and n_4 RCP $1.95\omega_0$ photons (perturbation). The emission frequency and spin of the channel (n_1, n_2, n_3, n_4) are given by

$$\Omega_{(n_1,n_2,n_3,n_4)} = (n_1 + n_3)\omega_0 + 1.95(n_2 + n_4)\omega_0 \quad \text{(IV.3)}$$

$$\sigma^{(0)}_{(n_1,n_2,n_3,n_4)} = -n_1 + n_2 + n_3 - n_4$$

Due to the perturbation photons (n_3, n_4), there exists allowed emission channels for all three spectral components of interest. Let us consider them one by one.

Harmonic 20.7 requires 6 photons of energy $1.95\omega_0$ and 9 photons of frequency ω_0. Its emission is enabled by the emission channel $(8,6,1,0)$ which requires one perturbation photon ($n_3 = 1$). Hence, to lowest order, it scales linearly with the strength of the perturbation (i.e. with the deviation angle).

Harmonic 19.75 requires 5 photons of energy $1.95\omega_0$ and 10 photons of frequency ω_0. There is no allowed emission channel that requires only one perturbation photon. However, the channel $(8,5,2,0)$ is allowed and requires 2 perturbation photons ($n_3 = 2$). Hence, to lowest order, it scales quadratically with the strength of the perturbation (i.e. with the deviation angle).

Harmonic 18.8 requires 4 photons of energy $1.95\omega_0$ and 11 photons of frequency ω_0. There are no allowed emission channels that require either one or two perturbation photons. However, there is a channel that requires three perturbation photons – $(8,4,3,0)$. This emission channel requires 3 perturbation photons hence it scales cubically (to lowest order) with the deviation angle.

Table IV.II:Consistency between emission-channel analysis and real-synthetic dynamical symmetry selection rule

Harmonic order	n_1 (pump)	n_2 (pump)	n_3 (perturbation)	n_4 (perturbation)	$\sigma_{(n_1,n_2,n_3,n_4)}$ (spin)	Number of required perturbation photons	Lowest order scaling predicted by real-synthetic symmetries
18.8	8	4	3	0	1	**3**	**Cubic**
19.75	8	5	2	0	1	**2**	**Quadratic**
20.7	8	6	1	0	1	**1**	**Linear**

Harmonic order $18.7\omega_0$ – selection rules, subchannels, and observation

In this subsection, we explore the scaling of the frequency component $18.7\omega_0$ as θ detunes from both 0° and 45°. We do so through real-synthetic symmetry selection rules (that is applicable in both regions) and by sub-channel analysis (which is applicable only around $\theta = 45°$). We show the consistency of the two approaches with each other, and with the experimental observation.

Real-synthetic symmetry analysis

By the real-synthetic selection rule of the symmetry $\hat{Z} \cdot \hat{\zeta}$ discussed in the main text, this frequency component is forbidden to have a linear $|\theta|$ dependence, hence it scales quadratically (to lowest order) as $|\theta|$ detunes from 0°.

We now consider the scaling of this frequency component as θ detunes from $45°$. Firstly, by the $\hat{C} \cdot \hat{\zeta}$ selection rule (III.5), the selection rule for $n = 18.7$ is

$$18.7 + 0.95 \times \ (a - b - c + d) \mp 1 = 0 \ (mod\ 2.95) \qquad \text{(IV.4)}$$

$$1 + 17.7 + 0.95 \times \ (a - b - c + d) \mp 1 = 0 \ (mod\ 2.95)$$

$$1 + 0.95 \times \ (a - b - c + d) \mp 1 = 0 \ (mod\ 2.95)$$

The condition (IV.4) is fulfilled by linear contributions such as $a = 1$ through $1 + 0.95 + 1 = 0 mod 2.95$. The condition is also fulfilled by quadratic contributions such as $a = b = 1$ through $1 + 0.95(1 - 1) - 1 = 0 mod 2.95$. Hence, both linear and quadratic contributions are allowed for the dependence of this frequency component with $\theta - 45°$.

<u>Sub-channel analysis</u>
Harmonic order $18.7\omega_0$ corresponds to the absorption of 7 fundamental ω_0 photons, and 6 second harmonic $1.95\omega_0$ photons. The emission of this frequency component is allowed at both $\theta = 0°$ and $\theta = 45°$ through the channel $(7,6,0,0)$ which requires no perturbation photons. As θ detunes from $45°$, H18.7 is also generated by the subchannels $(6,6,1,0)$ which requires 1 perturbation photon, and the subchannel $(6,5,1,1)$ which requires two perturbation photons. Hence, by sub-channel analysis, the scaling around $\theta = 45°$ exhibits both linear and quadratic contributions. This is consistent with the prediction obtained by real-synthetic symmetries, as well as with the observation (Figure IV.1).

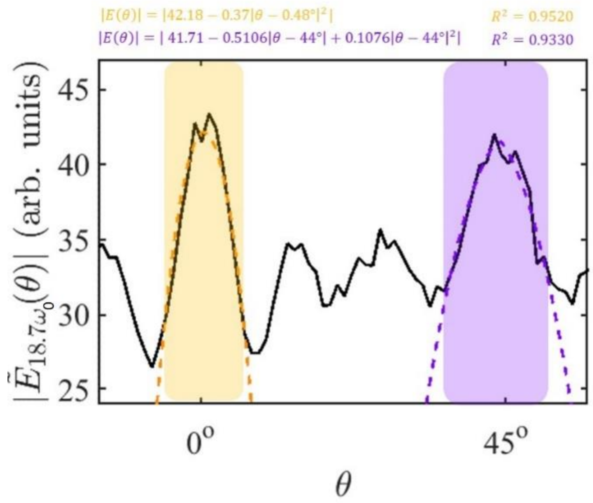

Figure IV.1: Scaling of harmonic order 18.7 *with the QWP angle* θ*, and comparison with prediction obtained from real-synthetic symmetries.*

Harmonic order $19.65\omega_0$ – selection rules, subchannels, and observation

In this subsection, we explore the scaling of the frequency component $19.65\omega_0$ as θ detunes from both $0°$ and $45°$. We do so through real-synthetic symmetry selection rules (that is applicable in both regions) and by sub-channel analysis (which is applicable only around $\theta = 45°$) We show the consistency of the two approaches with each other, and with the experimental observation.

Real-synthetic symmetry analysis

By the real-synthetic selection rule of the symmetry $\hat{Z} \cdot \hat{\zeta}$ discussed in the main text, this frequency component is forbidden to have a quadratic $|\theta - 0°|^2$ dependence, hence it scales linearly (to lowest order) as θ detunes from 0°.

We now consider the scaling of this frequency component as θ detunes from 45°. Firstly, by the $\hat{C} \cdot \hat{\zeta}$ selection rule (III.5), the selection rule for $n = 19.65$ is

$$19.65 + 0.95 \times \ (a - b - c + d) \mp 1 = 0 \ (mod\ 2.95) \qquad \text{(IV.5)}$$

$$1.95 + 17.7 + 0.95 \times \ (a - b - c + d) \mp 1 = 0 \ (mod\ 2.95)$$

$$1.95 + 0.95 \times \ (a - b - c + d) \mp 1 = 0 \ (mod\ 2.95)$$

The condition (IV.5) is fulfilled by linear contributions such as $b = 1$ through $1.95 - 0.95 - 1 = 0 mod 2.95$. The condition is also fulfilled by quadratic contributions such as $a = b = 1$ through $1.95 + 0.95(1 - 1) + 1 = 0 mod 2.95$. Hence, by real-synthetic symmetries, both linear and quadratic contributions are allowed for the dependence of this frequency component with $\theta - 45°$.

Sub-channel analysis

Harmonic order $19.65\omega_0$ corresponds to the absorption of 6 fundamental ω_0 photons, and 7 second harmonic $1.95\omega_0$ photons. The emission of this frequency component is allowed at both $\theta = 0°$ and $\theta = 45°$ through the channel $(6,7,0,0)$ which requires no perturbation photons. As θ detunes from 45°, H19.65 is also generated by the subchannels $(6,6,0,1)$ which requires 1 perturbation photon, and the subchannel $(5,6,1,1)$ which requires two perturbation photons. Hence, by sub-channel analysis, the scaling around $\theta = 45°$ exhibits both linear and quadratic contributions. This is consistent with the prediction obtained by real-synthetic symmetries, as well as with the observation (Figure IV.2).

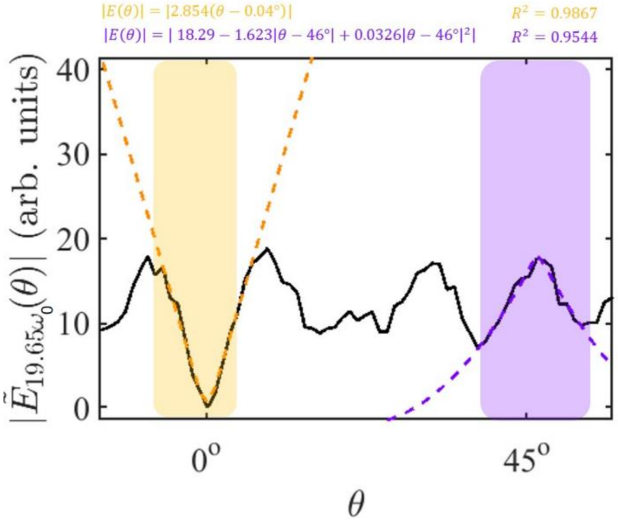

Figure IV.2: Scaling of harmonic order 19.65 *with the QWP angle* θ, *and comparison with prediction obtained from real-synthetic symmetries.*

Harmonic order $19.85\omega_0$ – selection rules, subchannels, and observation

In this subsection, we explore the scaling of the frequency component $19.65\omega_0$ as θ detunes from both 0° and 45°. We do so through real-synthetic symmetry selection rules (that is applicable in both regions) and by sub-channel analysis (which is applicable only around $\theta = 45°$). We show the consistency of the two approaches with each other, and with the experimental observation.

Real-synthetic symmetry analysis

By the real-synthetic selection rule of the symmetry $\hat{Z} \cdot \hat{\zeta}$ discussed in the main text, this frequency component is forbidden to have a quadratic $(\theta - 0°)$ dependence, hence it scales linearly (to lowest order) as θ detunes from 0°.

We now consider the scaling of this frequency component as θ detunes from 45°. Firstly, by the $\hat{C} \cdot \hat{\zeta}$ selection rule (III.5), the selection rule for $n = 19.85$ is

$$19.85 + 0.95 \times\ (a - b - c + d) \mp 1 = 0\ (mod\ 2.95) \qquad \text{(IV.6)}$$

$$2.15 + 2.95 \times 6 + 0.95 \times\ (a - b - c + d) \mp 1 = 0\ (mod\ 2.95)$$

$$2.15 + 0.95 \times\ (a - b - c + d) \mp 1 = 0\ (mod\ 2.95)$$

It is found that the condition (IV.6) is fulfilled (to lowest order in the perturbation) by $a = 5$ through $2.15 + 5 \times 0.95 - 1 = 5.9 = 0 mod 2.95$. It is also fulfilled by $a = 6$ through $2.15 + 6 \times 0.95 + 1 = 8.85 = 0m, od2.95$. Hence, by real-synthetic symmetries, this frequency component scales as a polynomial of fifth and sixth orders of $|\theta - 45°|$.

Sub-channel analysis

Harmonic order $19.85\omega_0$ corresponds to the absorption of 14 fundamental ω_0 photons, and 3 second harmonic $1.95\omega_0$ photons. The emission of this frequency component is forbidden at both $\theta = 0°$ and $\theta = 45°$. As θ detunes from 45°, this frequency component is generated by $(9,3,5,0)$ which requires 5 perturbation photons. Additionally, it is allowed by the subchannel $(8,3,6,0)$, which requires 6 perturbation photons.

Hence, by sub-channel analysis, the scaling around $\theta = 45°$ exhibits both fifth and sixth order contributions. This is consistent with the prediction obtained by real-synthetic symmetries, as well as with our experimental observation (Figure IV.3).

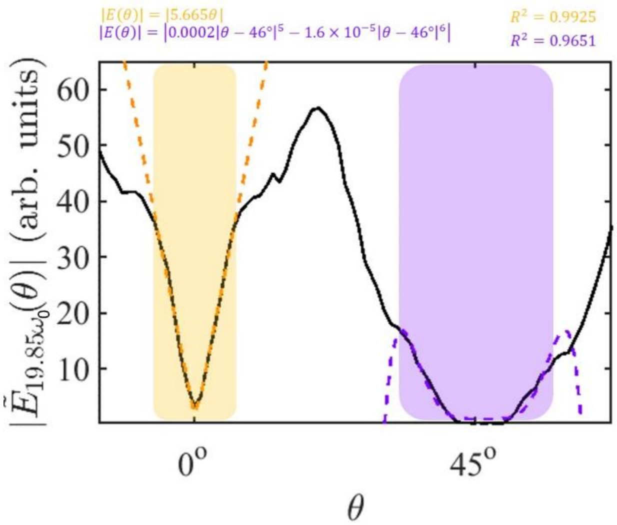

Figure IV.3: Scaling of harmonic order 19.85 *with the QWP angle* θ*, and comparison with prediction obtained from real-synthetic symmetries.*

V. Lissajous figures and ellipticity of the driving fields in the experimental configuration

In this section, we display the Lissajous curves of the driving field employed in the experimental investigation presented in the main text. Additionally, we provide the explicit expressions for $\epsilon(\theta)$, the ellipticity of the bi-elliptical drivers as a function of the waveplate angle.

Firstly, the driving field is given by

$$\mathbf{F}(\mathrm{t},\epsilon) = \sqrt{\frac{1}{1+\epsilon^2}}\Re\left\{e^{i\omega_0 t}(i\epsilon\hat{x}+\hat{y}) + \Delta e^{1.95 i\omega_0 t}(i\hat{x}-\epsilon\hat{y})\right\} \quad \text{(V.1)}$$

where ϵ is the ellipticity of the bi-elliptical drivers, $\hat{x}$ and $\hat{y}$ are cartesian basis vectors, ω_0 is the angular frequency of the fundamental beam (with 800nm wavelength), and $\Re$ symbolizes real part. Due to the non-integer frequency ratio, the period of this bi-chromatic field is $T = 20T_{\omega_0}$ where $T_{\omega_0} = 2\pi/\omega_0$ is the period of the fundamental field.

Figure V.I. depicts the Lissajous curves of the bi-chromatic driving field in the temporal range $0 < t < N_{cyc}T_{\omega_0}$ for various values of N_{cyc} and ϵ.

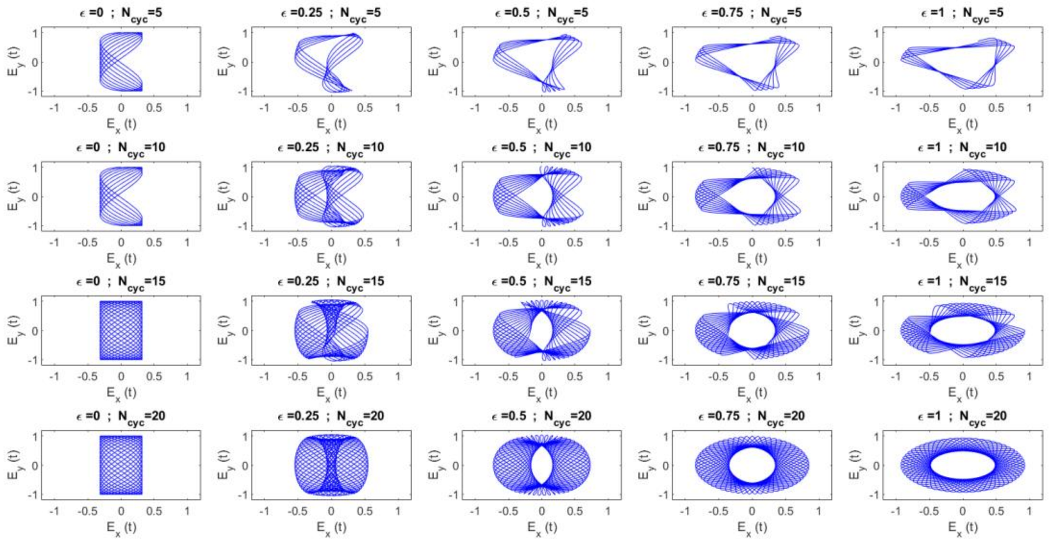

Figure V.1: Lissajous curves of the driving field used in our experiment (Eq.(V.I)) for different values of ϵ and N_{cyc} (number of T_{ω_0} cycles).

Next, we derive the ellipticity function $\epsilon(\theta)$ for the ellipticity of the bi-elliptical drivers ϵ as a function of the QWP angle θ. We consider a linearly polarized field of frequency ω_0 incoming into the QWP:

$$\mathbf{E}_{input}(t) = \left(e^{i\omega_0 t} + c.c.\right)\hat{x} \tag{V.2}$$

By Jones calculus[9], a QWP with an angle θ relative to the $\hat{x}$ axis transforms the polarization of the field to

$$\mathbf{E}_{\text{out}}(t,\theta) = e^{i\omega_0 t}(\cos^2(\theta) + isin^2(\theta))\hat{x} + e^{i\omega_0 t}(1-i)\sin(\theta)\cos(\theta)\,\hat{y} + c.c. \tag{V.3}$$

up to an overall phase. The $\hat{x}$ and $\hat{y}$ components of the complex amplitude of the output field are given by

$$E_x(\theta) = \cos^2(\theta) + isin^2(\theta) \tag{V.4}$$

$$E_y(\theta) = (1-i)\sin(\theta)\cos(\theta)\,\hat{y}$$

These complex amplitude components are used to evaluate the complex parameter χ:

$$\chi = \frac{E_y}{E_x} = \frac{(1-i)\sin(\theta)\cos(\theta)}{(\cos^2(\theta) + isin^2(\theta))} \tag{V.5}$$

The ellipticity of the field $\mathbf{E}_{\text{out}}(t,\theta)$ is obtained from χ by[10]

$$\epsilon = \tan\left(\frac{1}{2}\arcsin\left[-2\frac{Im\{\chi\}}{1+|\chi|^2}\right]\right) \tag{V.6}$$

where $Im\{\chi\}$ is the imaginary part of χ. Explicitly, this gives

$$\epsilon(\theta) = \tan\left(\frac{1}{2}\arcsin\left[-2\frac{Im\left\{\frac{(1-i)\sin(\theta)\cos(\theta)}{(\cos^2(\theta)+isin^2(\theta))}\right\}}{1+\left|\frac{(1-i)\sin(\theta)\cos(\theta)}{(\cos^2(\theta)+isin^2(\theta))}\right|^2}\right]\right) \quad \text{(V.7)}$$

More simply, this can be written as

$$\epsilon(\theta) = \tan\left(\left|\left(\theta-\frac{\pi}{4}\right)mod(\pi)-\frac{\pi}{2}\right|-\frac{\pi}{4}\right) \quad \text{(V.8)}$$

where *mod* is the modulo operation, and θ is given in radians. Notably, $\epsilon(\theta) = \tan(\theta)$ in the region $0 < \theta < \frac{\pi}{4}$.

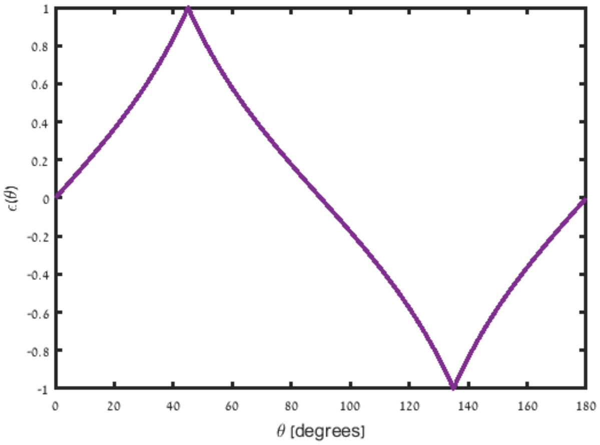

Figure V.2: the ellipticity of the bi-elliptical drivers $\epsilon(\theta)$ as a function of the quarter wave plate angle θ, given in degrees.

VI. Above threshold ionization (ATI) selection rules

Real-synthetic symmetries implicate selection rules on the above-threshold-ionization (ATI) spectra. In this section, we derive ATI selection rules, imposed by the real-synthetic symmetries that we derived in the previous section. The instantaneous ionization amplitude is given by

$$M_{\mathbf{k}}(t) = \langle \mathbf{k} | \phi_\alpha \rangle e^{-i\epsilon_\alpha t} \tag{VI.1}$$

where **k** is the momentum of the measured electron, $|\phi_\alpha\rangle$ is a resonant Floquet state and ϵ_α is the quasi-energy. The measured ionization is given by the cycle-average ionization amplitude

$$M_{\mathbf{k}} = \int_0^T \frac{dt}{T} \langle \mathbf{k} | \phi_\alpha(t) \rangle e^{-i\epsilon_\alpha t} \tag{VI.2}$$

Because $|\phi_\alpha(t)\rangle = |\phi_\alpha(t+T)\rangle$, the cycle-averaged ionization amplitude is non-zero for only specific values of **k**:

$$|\mathbf{k}_n\rangle = e^{i(\mathbf{k}\cdot\mathbf{r} - (\epsilon_\alpha + n\omega)t)} \tag{VI.3}$$

i.e., $|\mathbf{k}_\mathrm{n}\rangle$ corresponds to the energy $n\omega + \epsilon_\alpha \approx n\omega - I_p$.

$$M_{\mathbf{k}_\mathrm{n}} = \int_0^T \frac{dt}{T} e^{i(n\omega t - \boldsymbol{k}\cdot\boldsymbol{r})} \phi_\alpha(\mathbf{r}, t) \equiv \tilde{\phi}_{\alpha,\mathbf{k}_n} \tag{VI.4}$$

The cycle-averaged ionization amplitude is given by the spatio-temporal Fourier components $\phi_\alpha(\mathbf{r}, t)$, denoted by $\tilde{\phi}_{\alpha,\mathbf{k}_n}$. The measured ionization yield is given by $\left|M_{\mathbf{k}_n}\right|^2$. To obtain selection rules for the ionization amplitudes, one may expand $\tilde{\phi}_{\alpha,\boldsymbol{k}_n}$ as a power series of the perturbation parameters, (q_x, q_y).

$$M_{\mathbf{k}_n}(\mathbf{Q}) = \tilde{\phi}_{\alpha,\mathbf{k}_n}(\mathbf{Q}) = \sum_{\mathrm{a},b,c,d=0}^{\infty} \tilde{\phi}_{\mathbf{k}_n}^{(abcd)} \, q_x^{\mathrm{a}} q_y^{\mathrm{b}} \bar{q}_x^{\mathrm{c}} \bar{q}_y^{\mathrm{d}} \tag{VI.5}$$

$$|\phi\rangle = \sum_{\substack{\mathrm{a},b,c,d=0 \\ \mathbf{k}_n}}^{\infty} \tilde{\phi}_{\alpha,\mathbf{k}_n}^{(abcd)} e^{i\mathbf{k}\cdot\mathbf{r}} e^{in\omega t} \, q_x^{\mathrm{a}} q_y^{\mathrm{b}} \bar{q}_x^{\mathrm{c}} \bar{q}_y^{\mathrm{d}} \tag{VI.6}$$

and apply the relevant synthetic symmetry operation.

$\hat{\boldsymbol{Z}}_y = \hat{\boldsymbol{\tau}}_2 \cdot \hat{\boldsymbol{\sigma}}_y$ symmetry

In this subsection, we derive the selection rules for a $\hat{Z}_y$ symmetric system, perturbed by a laser with a polarization **Q** and frequency $s\omega$. The perturbation imposes on the system the symmetry $\hat{Z}_y \cdot \hat{\zeta}_{\hat{Z}_y}$ where

$$\hat{\zeta}(\mathbf{Q}) = \begin{pmatrix} (-1)^{s+1} & 0 \\ 0 & (-1)^s \end{pmatrix} \begin{pmatrix} q_x \\ q_y \end{pmatrix} \tag{VI.7}$$

We operate with $\hat{Z}_y \cdot \hat{\zeta}_{\hat{Z}_y}$ on $|\phi\rangle$,

$$\hat{Z}_y \cdot \hat{\zeta}_{\hat{Z}_y}|\phi\rangle = \sum_{\substack{a,b,c,d=0 \\ \mathbf{k}_n}}^{\infty} \tilde{\phi}_{\alpha,\mathbf{k}_n}^{(abcd)} e^{i\mathbf{k}\cdot(\hat{\sigma}_y\cdot\mathbf{r})} e^{in\omega t} (-1)^{n+(s+1)(a+c)+s(b+d)} q_x^a q_y^b \bar{q}_x^c \bar{q}_y^d \qquad \text{(VI.8)}$$

Because $\hat{Z}_y \cdot \hat{\zeta}_{\hat{Z}_y}$ is a symmetry of the system, $|\phi\rangle$ is an eigenfunction of it with an eigenvalue of either +1 or -1. Assuming that it has an eigenvalue of +1, we obtain the following restriction on the emission:

$$\tilde{\phi}_{\alpha,\mathbf{k}_n}^{(abcd)} = \tilde{\phi}_{\alpha,\hat{\sigma}_y\mathbf{k}_n}^{(abcd)} (-1)^{n+(s+1)(a+c)+s(b+d)} \qquad \text{(VI.9)}$$

An analogous expression may be obtained for states whose eigenvalue is -1. To illustrate what this restriction means physically, we consider an example. When ATI is driven by an $\omega - 2\omega$ cross linear driving field,

$$E(t) = \begin{pmatrix} \sin(\omega t) \\ \cos(2\omega t) \end{pmatrix} \qquad \text{(VI.10)}$$

the system exhibits $\hat{Z}_y$ DS. Thus, along the $\hat{y}$ axis (i.e. when the detector is situated on the polarization axis of the 2ω pump field), only even order photoelectron peaks are allowed [11]. If the system is perturbed by the field $\lambda \cos(2\omega t)\,\hat{x}$, the symmetry is broken and odd order photoelectron peaks are generated along the $\hat{y}$ axis. We plug in $s = 2, b = d = 0$, and obtain the following restriction on the photoemission spectrum

$$\tilde{\phi}_{\alpha,\mathbf{k}_n}^{(a0c0)} = \tilde{\phi}_{\alpha,\hat{\sigma}_y\mathbf{k}_\mathrm{n}}^{(a0c0)} (-1)^{n+a+c} \qquad \text{(VI.11)}$$

For electrons whose momentum is parallel to the $\hat{y}$ axis (perpendicular to the perturbation),

$$\mathbf{k}_\mathrm{n} = \hat{\sigma}_y \mathbf{k}_\mathrm{n} \qquad \text{(VI.12)}$$

and thus

$$\tilde{\phi}_{\alpha,\mathbf{k}_\mathrm{n}}^{(a0c0)} = \tilde{\phi}_{\alpha,\mathbf{k}_\mathrm{n}}^{(a0c0)} (-1)^{n+a+c} \qquad \text{(VI.13)}$$

i.e. $\tilde{\phi}_{\alpha,\mathbf{k}_\mathrm{n}}^{(abcd)}$ is only nonzero if n and $(a + c)$ are of the same parity. Thus, along the $\hat{y}$ axis, even ordered photoelectron peaks scale as even orders of the perturbation's amplitude and odd order photoelectron peaks scale as odd powers of the perturbation's amplitude.

$\hat{\boldsymbol{C}}_{\boldsymbol{NM}} = \hat{\boldsymbol{R}}_{\boldsymbol{N,M}} \cdot \hat{\boldsymbol{\tau}}_{\boldsymbol{N}}$ symmetry

When a monochromatic laser electric field breaks $\hat{C}_{NM}$ symmetry, the system exhibits the reduced symmetry $\hat{C}_{N,M} \cdot \hat{\zeta}_{\hat{C}_{N,M}}$ where

$$\hat{\zeta}_{\hat{C}_{N,M}}(\mathbf{Q}) = e^{-\frac{i2\pi s}{N}} \hat{R}^{(\boldsymbol{Q})}{}_{N,M} \cdot \mathbf{Q} \qquad \text{(VI.14)}$$

We expand $|\phi(\mathbf{r},\mathbf{Q},t)\rangle$ to a power series in terms of the RCP and LCP components of the perturbation

$$|\phi\rangle = \sum_{\substack{\mathrm{a,b,c,d}=0 \\ n,\mathbf{k}}}^{\infty} \tilde{\phi}_{\mathbf{k}n}{}^{(abcd)} e^{-i\boldsymbol{k}\cdot\boldsymbol{r}} e^{in\omega t}\, q_R^{\mathrm{a}} q_L^{\mathrm{b}} \bar{q}_R^{\mathrm{c}} \bar{q}_L^{\mathrm{d}} \tag{VI.15}$$

where $q_{R,L} = q_x \mp i q_y$ are the RCP and LCP polarization components of $\mathbf{Q}$. The operation of $e^{-\frac{i2\pi s}{N}} \hat{R}_{NM}^{(Q)}$ on $\mathbf{Q}$ results in $q_- \to e^{\frac{i2\pi}{N}(-s-M)} q_-$ and $q_+ \to e^{\frac{i2\pi}{N}(-s+M)} q_+$.

Operating with $\hat{C}_{NM} \cdot \hat{\zeta}_{\hat{C}_{N,M}}$ on $|\phi\rangle$:

(VI.16)

$$\hat{C}_{NM} \cdot \hat{\zeta}_{\hat{C}_{N,M}} |\phi\rangle = \sum_{\substack{\mathrm{a,b,c,d}=0 \\ n,\boldsymbol{k}}}^{\infty} \tilde{\phi}_{\boldsymbol{k}n}{}^{(abcd)} e^{-i\mathbf{k}\cdot(\hat{R}_{NM}\cdot\mathbf{r})} e^{in\omega t}\, e^{i\frac{2\pi n}{N}} e^{\frac{i2\pi}{N}(-s-M)(a-c)} e^{\frac{i2\pi}{N}(-s+M)(b-d)} q_R^{\mathrm{a}} q_L^{\mathrm{b}} \bar{q}_R^{\mathrm{c}} \bar{q}_L^{\mathrm{d}}$$

Thus

$$\tilde{\phi}_{\mathbf{k}_n}{}^{(abcd)} = \tilde{\phi}_{\hat{R}_{NM}\boldsymbol{k}_n}{}^{(abcd)} e^{i\frac{2\pi}{N}[n+(M-s)(a-c)-(M+s)(b-d)]} \tag{VI.17}$$

$\hat{\boldsymbol{e}}_{\boldsymbol{NM}} = \hat{\boldsymbol{R}}_{\boldsymbol{N,M}} \cdot \hat{\boldsymbol{\tau}}_{\boldsymbol{N}}$ symmetry

When a monochromatic laser electric field breaks $\hat{e}_{NM}$ symmetry, the system exhibits the synthetic symmetry $\hat{e}_{N,M} \cdot \check{\zeta}_{\hat{e}_{NM}}$ where

$$\hat{\zeta}_{\hat{e}_{NM}}(\mathbf{Q}) = e^{-\frac{i2\pi s}{N}} \hat{L}^{(\mathbf{Q})}{}_{1/b} \cdot \hat{R}^{(\mathbf{Q})}{}_{N,M} \cdot \hat{L}^{(\mathbf{Q})}{}_{b} \tag{VI.18}$$

We expand $|\phi(\mathbf{r},\mathbf{Q},t)\rangle$ in to a power series in right-handed and left-handed elliptical polarization components of $\mathbf{Q}$, defined by $q_{\mp} = q_x \mp i b q_y$. The series expansion is given by:

$$|\phi\rangle = \sum_{\substack{\mathrm{a,b,c,d}=0 \\ n,\mathbf{k}}}^{\infty} \tilde{\phi}_{\mathbf{k}n}{}^{(abcd)} e^{-i\mathbf{k}\cdot\mathbf{r}} e^{in\omega t}\, q_-^{\mathrm{a}} q_+^{\mathrm{b}} \bar{q}_-^{\mathrm{c}} \bar{q}_+^{\mathrm{d}} \tag{VI.19}$$

The operation of $e^{-\frac{i2\pi s}{N}} \hat{L}^{(\mathbf{Q})}{}_{1/b} \cdot \hat{R}^{(\mathbf{Q})}{}_{N,M} \cdot \hat{L}^{(\mathbf{Q})}{}_{b}$ on $\mathbf{Q}$ results in $q_- \to e^{\frac{i2\pi}{N}(-s+M)} q_-$ and $q_+ \to e^{\frac{i2\pi}{N}(-s-M)} q_+$.

(VI.20)

$$\hat{e}_{N,M}\cdot\hat{\zeta}_{\hat{e}_{NM}}|\phi\rangle = \sum_{\substack{a,b,c,d=0\\ n,\mathbf{k}}}^{\infty}\tilde{\phi}_{\mathbf{k}n}^{(abcd)}e^{-i\mathbf{k}\cdot(\hat{L}_b\cdot\hat{R}_{N,M}\cdot\hat{L}_{1/b}\cdot\boldsymbol{r})}e^{in\omega t}\,e^{i\frac{2\pi n}{N}}e^{\frac{i2\pi}{N}(-s-M)(a-c)}e^{\frac{i2\pi}{N}(-s+M)(b-d)}\mathrm{q}_-^{\mathrm{a}}\mathrm{q}_+^{\mathrm{b}}\bar{\mathrm{q}}_-^{\mathrm{c}}\bar{\mathrm{q}}_+^{\mathrm{d}}$$

Thus

$$\tilde{\phi}_{\mathbf{k}_n}^{(abcd)} = \tilde{\phi}_{\hat{L}_b\cdot\hat{R}_{N,M}\cdot\hat{L}_{1/b}\mathbf{k}_n}^{(abcd)}e^{i\frac{2\pi}{N}[n+(-M-s)(a-c)-(-M+s)(b-d)]} \qquad \text{(VI.21)}$$

$\hat{\mathbf{T}}$ symmetry

When a monochromatic laser electric field breaks $\hat{T}$ symmetry, the system exhibits the synthetic symmetry $\hat{T}\cdot\hat{\zeta}_{\hat{T}}$ where

$$\hat{\zeta}_{\hat{T}}(\mathbf{Q}) = \bar{\mathbf{Q}} \qquad \text{(VI.22)}$$

Operating with $\hat{T}\cdot\hat{\zeta}_{\hat{T}}$ on $|\phi\rangle$ we obtain

$$\hat{T}\cdot\hat{\zeta}_{\hat{T}}|\phi\rangle = \sum_{\substack{abcd=0\\ n,\mathbf{k}}}^{\infty}\tilde{\phi}_{\mathbf{k},-n}^{(cdab)}e^{i\mathbf{k}\cdot\mathbf{r}}e^{in\omega t}\,q_{\mathrm{x}}^{\mathrm{a}}q_y^{\mathrm{b}}\bar{q}_x^{\mathrm{c}}\bar{q}_y^{\mathrm{d}} \qquad \text{(VI.23)}$$

We obtain the selection rule:

$$\tilde{\phi}_{\mathbf{k}n}^{(abcd)} = \tilde{\phi}_{\mathbf{k},-n}^{(cdab)} \qquad \text{(VI.24)}$$

$\hat{\mathbf{Q}}$ symmetry

When a monochromatic laser electric field breaks $\hat{Q}$ symmetry, the system exhibits the synthetic symmetry $\hat{Q}\cdot\hat{\zeta}_{\hat{Q}}$ where

$$\hat{\zeta}_{\hat{Q}}(\mathbf{Q}) = -\bar{\mathbf{Q}} \qquad \text{(VI.25)}$$

Operating with the synthetic symmetry operation on the Floquet state, we obtain

$$\hat{T}\cdot\hat{R}_2\cdot\hat{\zeta}_{\hat{Q}}|\phi\rangle = \sum_{\substack{a,b,c,d=0\\ n,\boldsymbol{k}}}^{\infty}\tilde{\phi}_{\mathbf{k}n}^{(cdab)}e^{i\mathbf{k}\cdot\hat{R}_2\boldsymbol{r}}e^{-in\omega t}\,(-1)^{a+b+c+d}\,q_{\mathrm{x}}^{\mathrm{a}}q_y^{\mathrm{b}}\bar{q}_x^{\mathrm{c}}\bar{q}_y^{\mathrm{d}} \qquad \text{(VI.26)}$$

which results in the selection rule

$$\tilde{\phi}_{\mathbf{k}n}^{(abcd)} = (-1)^{a+b+c+d}\tilde{\phi}_{(-\boldsymbol{k})(-n)}^{(cdab)} \qquad \text{(VI.27)}$$

***$\hat{G}$* symmetry**

When a monochromatic laser electric field breaks $\hat{G}$ symmetry, the system exhibits the synthetic symmetry $\hat{G}\cdot\hat{\zeta}_{\hat{G}}$ where

$$\hat{\zeta}_{\hat{G}}(\mathbf{Q}) = (-1)^{s+1}\overline{\mathbf{Q}} \tag{VI.28}$$

Operating with $\hat{G}\cdot\hat{\zeta}_{\hat{G}}$ on $|\phi\rangle$ we obtain

$$\hat{T}\cdot\hat{\tau}_2\cdot\hat{R}_2\cdot\hat{\zeta}_{\hat{G}}|\phi\rangle = \sum_{\substack{a,b,c,d=0\\ n,\mathbf{k}}}^{\infty} \tilde{\phi}_{\mathbf{k}n}{}^{(cdab)} e^{i\mathbf{k}\cdot\hat{R}_2 r} e^{-in\omega t}\,(-1)^{n+(s+1)(a+b+c+d)}\; q_{\mathrm{x}}^{\mathrm{a}} q_y^{\mathrm{b}} \bar{q}_x^{\mathrm{c}} \bar{q}_y^{\mathrm{d}} \tag{VI.29}$$

which results in the equation

$$\tilde{\phi}_{\mathbf{k}n}{}^{(abcd)} = \tilde{\phi}_{(-\mathbf{k})(-n)}{}^{(cdab)}(-1)^{n+(s+1)(a+b+c+d)} \tag{VI.30}$$

$\widehat{\boldsymbol{D}}_{\boldsymbol{y}}$ symmetry

When a monochromatic laser electric field breaks $\widehat{D}_y$ symmetry, the system exhibits the synthetic symmetry $\widehat{D}_y\cdot\check{\zeta}_{\widehat{D}_y}$ where

$$\hat{\zeta}_{\widehat{D}_y}(\mathbf{Q}) = \begin{pmatrix} -1 & 0 \\ 0 & 1 \end{pmatrix}\begin{pmatrix} \bar{q}_x \\ \bar{q}_y \end{pmatrix} \tag{VI.31}$$

Operating with $\widehat{D}_y\cdot\hat{\zeta}_{\widehat{D}_y}$ on $|\phi\rangle$ results in

$$\widehat{D}_y\cdot\hat{\zeta}_{\widehat{D}_y}|\phi\rangle = \sum_{\substack{a,b,c,d,=0\\ n,\mathbf{k}}}^{\infty} \tilde{\phi}_{\boldsymbol{k}n}{}^{(cdab)} e^{i\mathbf{k}\cdot\hat{\sigma}_y\mathbf{r}} e^{-in\omega t}\,(-1)^{(a+c)}\; q_{\mathrm{x}}^{\mathrm{a}} q_y^{\mathrm{b}} \bar{q}_x^{\mathrm{c}} \bar{q}_y^{\mathrm{d}} \tag{VI.32}$$

which results in the selection rule

$$\tilde{\phi}_{\mathbf{k}n}{}^{(cdab)} = \tilde{\phi}_{(\hat{\sigma}_y\mathbf{k})(-n)}{}^{(abcd)}(-1)^{(a+c)} \tag{VI.33}$$

$\widehat{H}_y$ symmetry

When a monochromatic laser electric field breaks $\widehat{H}_y$ symmetry, the system exhibits the synthetic symmetry operation $\widehat{H}_y\cdot\hat{\zeta}_{\widehat{H}_y}$ where

$$\hat{\zeta}_{\widehat{H}_y}(\mathbf{Q}) = \begin{pmatrix} (-1)^{s+1} & 0 \\ 0 & (-1)^{s} \end{pmatrix}\begin{pmatrix} \bar{q}_x \\ \bar{q}_y \end{pmatrix} \tag{VI.34}$$

Operating with $\widehat{H}\cdot\hat{\zeta}_{\widehat{H}_y}$ on $|\phi\rangle$ results in

$$\hat{T}\cdot\hat{\tau}_2\cdot\hat{\sigma}_y\cdot\hat{\zeta}_{\hat{H}_y}|\phi\rangle = \sum_{\substack{a,b,c,d=0\\ n,\boldsymbol{k}}}^{\infty}\tilde{\phi}_{\boldsymbol{k}n}{}^{(c,dab)}e^{i\boldsymbol{k}\cdot\hat{\boldsymbol{\sigma}}_y\boldsymbol{r}}e^{-in\omega t}\,(-1)^{n+(s+1)(a+c)+s(b+d)}\,q_x^a q_y^b \bar{q}_x^c \bar{q}_y^d \tag{VI.35}$$

which results in

$$\tilde{\phi}_{\mathbf{k}n}{}^{(abcd)} = \tilde{\phi}_{\sigma_y\mathbf{k},-n}{}^{(cdab)}(-1)^{n+(s+1)(a+c)+s(b+d)} \tag{VI.36}$$

Supplementary table VI.I: Photoionization selection rules through real-synthetic symmetries

$\hat{X}$	$\hat{\zeta}_{\hat{X}}(\mathbf{Q})$	Above threshold ionization selection rule
$\hat{T}$	$\bar{\mathbf{Q}}$	$\tilde{\phi}_{\mathbf{k}n}{}^{(abcd)} = \tilde{\phi}_{\mathbf{k},-n}{}^{(cdab)}$
$\hat{Q}$	$-\bar{\mathbf{Q}}$	$\tilde{\phi}_{\mathbf{k}n}{}^{(abcd)} = (-1)^{a+b+c+d}\tilde{\phi}_{(-\mathbf{k})(-n)}{}^{(cdab)}$
$\hat{G}$	$(-1)^{1+s}\bar{\mathbf{Q}}$	$\tilde{\phi}_{\mathbf{k}n}{}^{(abcd)} = \tilde{\phi}_{(-\mathbf{k})(-n)}{}^{(cdab)}(-1)^{n+(s+1)(a+b+c+d)}$
$\hat{Z}_y$	$\begin{pmatrix}(-1)^{s+1} & 0\\ 0 & (-1)^s\end{pmatrix}\begin{pmatrix}q_x\\ q_y\end{pmatrix}$	$\tilde{\phi}_{\alpha,\mathbf{k}_n}^{(abcd)} = \tilde{\phi}_{\alpha,\hat{\sigma}_y\mathbf{k}_\mathrm{n}}^{(abcd)}(-1)^{n+(s+1)(a+c)+s(b+d)}$
$\hat{D}_y$	$\begin{pmatrix}-1 & 0\\ 0 & 1\end{pmatrix}\begin{pmatrix}\bar{q}_x\\ \bar{q}_y\end{pmatrix}$	$\tilde{\phi}_{\mathbf{k}n}{}^{(cdab)} = \tilde{\phi}_{(\hat{\sigma}_y\mathbf{k})(-n)}{}^{(abcd)}(-1)^{(a+c)}$
$\hat{H}_y$	$\begin{pmatrix}(-1)^{s+1} & 0\\ 0 & (-1)^s\end{pmatrix}\begin{pmatrix}\bar{q}_x\\ \bar{q}_y\end{pmatrix}$	$\tilde{\phi}_{\mathbf{k}n}{}^{(abcd)} = \tilde{\phi}_{\sigma_y\mathbf{k},-n}{}^{(cdab)}(-1)^{n+(s+1)(a+c)+s(b+d)}$
$\hat{C}_{NM}$	$e^{-\frac{i2\pi s}{N}}\hat{R}^{(\mathbf{Q})}{}_{N,M}\cdot\mathbf{Q}$	$\tilde{\phi}_{\mathbf{k}_n}{}^{(abcd)} = \tilde{\phi}_{\hat{R}_{NM}\mathbf{k}_n}{}^{(abcd)}e^{i\frac{2\pi}{N}[n+(M-s)(a-c)-(M+s)(b-d)]}$
$\hat{e}_{NM}$	$e^{-\frac{i2\pi s}{N}}\hat{L}^{(\mathbf{Q})}{}_{1/b}\cdot\hat{R}^{(\mathbf{Q})}{}_{N,M}\cdot\hat{L}^{(\mathbf{Q})}{}_b\cdot\mathbf{Q}$	$\tilde{\phi}_{\mathbf{k}_n}{}^{(abcd)} = \tilde{\phi}_{\hat{L}_b\cdot\hat{R}_{N,M}\cdot\hat{L}_{1/b}\mathbf{k}_n}{}^{(abcd)}e^{i\frac{2\pi}{N}[n+(M-s)(a-c)-(M+s)(b-d)]}$

VII. Synthetic dynamical symmetries in spin orbit coupled systems

In this section, we present an example of real-synthetic symmetries imposed by Rashba and Dresselhaus spin-orbit coupling strengths. We consider the following tight-binding Hamiltonian[12] of a lattice under an external magnetic field:

$$\hat{H} = \sum_{\mathbf{k}} \Psi_{\mathbf{k}}^{\dagger}\{\epsilon(\mathbf{k}) \otimes \sigma_0 - [\alpha \sin(k_y a) - \gamma \sin(k_x a)] \otimes \sigma_x + [\alpha \sin(k_x a) - \gamma \sin(k_y z)] \otimes\times \sigma_y + B\sigma_z\}\Psi_{\boldsymbol{k}} \tag{VII.1}$$

where $\Psi_{\mathbf{k}} = (\hat{c}_{\mathbf{k}\uparrow}, \hat{c}_{\mathbf{k}\downarrow})^T$is a spinor combining the annihilation/creation operators for a fermion with momentum $\mathbf{k}$, $\epsilon(\mathbf{k}) = 2t_h[4 - \cos(k_x a) - \cos(k_y a)]$ is the dispersion of the lattice, σ_0 is the identity matrix, $\sigma_{x,y,z}$ are the pauli matrices, and t_h is a real parameter. The coefficients α and γ are the spin-orbit coupling strengths, for the Rashba and Dressehauls couplings, respectively. B is an external magnetic field which is assumed to couple only to the spin.

This model was discussed in the context of HHG in spin-orbit coupled systems[8], as well as in other physical contexts[13–15].

Circularly polarized driving field

Let us assume that the model presented above is driven by a circularly polarized laser whose vector potential is denoted by $\mathbf{A}(t)$. The effect of a driving field may be incorporated in the model by a Peierls substitution

$$\mathbf{k}(t) = \mathbf{k}(0) + \mathbf{A}(t) \tag{VII.2}$$

For a circularly polarized driver with amplitude of frequency Ω, the vector potential $\boldsymbol{A}(t)$ is given by

$$\mathbf{A}(t) = A_0 \begin{pmatrix} \cos(\Omega t) \\ \sin(\Omega t) \\ 0 \end{pmatrix} \tag{VII.3}$$

For a Rashba model ($\gamma = 0$), one may employ the spin of the system as a synthetic dimension and obtain a synthetic symmetry operation of order 4. The driven Rashba Hamiltonian exhibits the symmetry[12]

$$\hat{X} \equiv \hat{R}_4 \cdot \hat{\tau}_4 \cdot \hat{S}_4 \tag{VII.4}$$

where $\hat{R}_4$ and $\hat{S}_4$ are $\frac{2\pi}{4}$ rotations in real space and spin space respectively, and $\hat{\tau}_4$ is a time translation by $\frac{T}{4}$ where $T = \frac{2\pi}{\Omega}$. Explicitly, $\hat{X}$ is given by

$$\hat{R}_4 \cdot \hat{\tau}_4 \cdot \hat{S}_4 = \begin{cases} (k_x, k_y) \to (k_y, -k_x) \\ t \to t + \dfrac{T}{4} \\ (\sigma_x, \sigma_y, \sigma_z) \to (+\sigma_y, -\sigma_x, \sigma_z) \end{cases} \tag{VII.5}$$

The corresponding HHG selection rule determines that only $4n-1$ $(4n+1)$ harmonics are allowed with right (left) handed circular polarization. For a Rashba-Dresselhaus model ($\alpha \neq \gamma \neq 0$), the symmetry $\hat{X}$ and its corresponding selection rules are broken, and there is no symmetry corresponding to a $T/4$ time translation. However, it is possible to exploit the broken symmetry to formulate a synthetic symmetry of the form $\hat{X} \cdot \hat{\zeta}_{\hat{X}}$, where $\hat{\zeta}_{\hat{X}}$ operates on the synthetic γ dimension. The driven Rashba-Dresselhaus model exhibits the symmetry $\hat{X} \cdot \hat{\zeta}$ where $\hat{X}$ is given in Eq. (VII.5) and $\hat{\zeta}_{\hat{X}}(\gamma) = -\gamma$.

Next, we derive the HHG selection rules. The standard selection rules due to DS $\hat{X}$ was derived in Ref[12]. We extend their treatment to real-synthetic symmetries of the form $\hat{X} \cdot \hat{\zeta}$. The HHG spectrum is given by the expression $|i\omega J_i(\omega)|^2$ where $|J_i(\omega)|$ is Fourier transform of the charge current, and the magnetization current was neglected. The charge current is given by $J_i(t) = N_k^{-1} \sum_{\mathbf{k}} Tr[\rho(t) v_i(\mathbf{k}, t)]$ in the $i = x, y, z$ direction. Here, $\rho(t)$ is the time dependent charge density and $v_i(\mathbf{k}, t)$ is the charge velocity. Generally, to obtain selection rules on the complete harmonic emission, one needs operate on $J_i(t)$ with $\hat{X} \cdot \hat{\zeta}$ and employ its invariance under the symmetry operation. While this is possible, Ref[12] showed that for this model, the selection rules are well approximated by the emission of a single electron in a well-defined crystal momentum $\boldsymbol{k}$. Hence, for simplicity, we derive the selection rules for $\mathbf{v}(\mathbf{k}, t)$ and not $\mathbf{J}(t)$. We first express the charge velocity $\mathbf{v}(\mathbf{k}, t)$ using a circularly polarized basis, that is, we solve for the selection rules of

$$v_{\pm}(\mathbf{k}, t) = v_x(\mathbf{k}, t) \pm i v_y(\mathbf{k}, t) \tag{VII.6}$$

The driven Hamiltonian is explicitly given by

$$\hat{H} = \sum_{\mathbf{k}} \Psi_{\mathbf{k}}^{\dagger} h(\mathbf{k}, t) \Psi_{\mathbf{k}} \tag{VII.7}$$

$$\begin{aligned} h(\mathbf{k}, t) = & \left[8 - 2\cos\left(k_{x0} + A_x(t)\right) - 2\cos\left(k_{y0} + A_y(t)\right)\right] \otimes \sigma_0 \\ & - \left[\alpha \sin\left(k_{y0} + A_y(t)\right) - \gamma \sin\left(k_{x0} + A_x(t)\right)\right] \otimes \sigma_x \\ & + \left[\alpha \sin\left(k_{x0} + A_x(t)\right) - \gamma \sin\left(k_{y0} + A_y(t)\right)\right] \otimes \sigma_y + B\sigma_z \end{aligned}$$

The Cartesian components of the velocity are given by

$$v_x = \frac{\partial h(\mathbf{k},t)}{\partial k_x} = [2\sin(k_{x0} + A_x(t))] \otimes \sigma_0 + [\gamma \cos(k_{x0} + A_x(t))] \otimes \sigma_x + [\alpha \cos(k_{x0} + A_x(t))] \otimes \sigma_y \tag{VII.8}$$

$$v_y = \frac{\partial h(\mathbf{k},t)}{\partial k_y} = \left[2\sin\left(k_{y0} + A_y(t)\right)\right] \otimes \sigma_0 - \left[\alpha \cos\left(k_{y0} + A_y(t)\right)\right] \otimes \sigma_x - \left[\gamma \cos\left(k_{y0} + A_y(t)\right)\right] \otimes \sigma_y$$

Hence, $v_{\pm}(\mathbf{k},t)$ are given by

$$\begin{aligned} v_{\pm} = v_x \pm i v_y = 2\left[\sin(k_{x0} + A_x(t)) \pm i \sin\left(k_{y0} + A_y(t)\right)\right] \otimes \sigma_0 \\ + \left[\gamma \cos(k_{x0} + A_x(t)) \mp i\alpha \cos\left(k_{y0} + A_y(t)\right)\right] \otimes \sigma_x \\ + \left[\alpha \cos(k_{x0} + A_x(t)) \mp i\gamma \cos\left(k_{y0} + A_y(t)\right)\right] \otimes \sigma_y \end{aligned} \tag{VII.9}$$

The amplitude of the nth harmonic can be obtained from $v_{\pm}(\mathbf{k},t)$ by a Fourier transform. We operate with $\hat{X} \cdot \hat{\zeta}_{\hat{X}}$ on Eq. (VII.9), and obtain the following conditions on the polarization components of the harmonic n:

$$e^{\frac{in\pi}{2}} E_{n\pm}(\gamma) = \pm i E_{n\pm}(-\gamma) \tag{VII.10}$$

Explicitly, this translates to the following selection rules:

$$E_{4n+1,+}(\gamma) = E_{4n+1,+}(\gamma) \tag{VII.11}$$

$$E_{4n-1,+}(\gamma) = -E_{4n-1,+}(-\gamma) \tag{VII.12}$$

$$E_{(4n-1),-}(\gamma) = E_{(4n-1),-}(-\gamma) \tag{VII.13}$$

$$E_{(4n+1),-}(\gamma) = -E_{(4n+1),-}(-\gamma) \tag{VII.14}$$

$$\mathbf{E}_{2n}(\gamma) = 0 \tag{VII.15}$$

That is, the synthetic dynamical symmetry $\hat{X} \cdot \hat{\zeta}_{\hat{X}}$ forbids even harmonic generation, even though the dynamical symmetry $\hat{X}$ is broken. Furthermore, the symmetry $\hat{X} \cdot \hat{\zeta}_{\hat{X}}$ restricts the dependence of harmonics $E_{4n\pm1,\pm}(\gamma)$ to be either odd or even in γ, the spin-orbit coupling strength.

VIII. Dark states and bands by real-synthetic symmetries

In this section, we demonstrate how the concept of real-synthetic symmetries may be used to obtain selection rules for dark states & bands in periodically driven Floquet systems[4,16]. Consider a material sample subject to a pump field of frequency Ω and polarization $\mathbf{p}_{\boldsymbol{\Omega}}$ that dresses the sample, changing its absorption properties. A probe field of variable frequency ω_p and polarization $\mathbf{p}_{\omega_p}$ is used to probe the altered absorption and emission properties of the dressed sample. The Hamiltonian of the system is given by

$$\hat{H} = \underbrace{\hat{H}_{Material} + \Re\{\mathbf{p}_\Omega \cdot \hat{\mu} e^{i\Omega t}\}}_{\hat{H}_0} + \underbrace{\Re\left\{\mathbf{p}_{\omega_p} \cdot \mathbf{r} e^{i\omega_p t}\right\}}_{\hat{W}} \tag{VIII.1}$$

where $\hat{H}_0$ is the Hamiltonian of the dressed sample without the probe field, and $\hat{W}$ stands for the probe field. As previously exemplified[4] using perturbation theory, DSs in $\hat{H}_0$ result in selection rules on the absorption of the probe, such as symmetry-protected dark states, symmetry protected dark-bands, and symmetry-induced transparency. In the following, we will demonstrate using real-synthetic symmetries our approach extends the previously derived rules to beyond the regime of linear response.

For example, consider a benzene ring driven by a circularly polarized laser from above, so that its Hamiltonian is given by

$$\hat{H}_0 = \hat{H}_B(x, y) + E_0 \Re\{(x - iy)e^{i\Omega t}\} \tag{VIII.2}$$

where $\hat{H}_B(x, y)$ is the Hamiltonian of the field free benzene ring. The Hamiltonian $\hat{H}_0(x, y, t)$ exhibits $\hat{C}_6 = \hat{\tau}_6 \cdot \hat{R}_6^{(z)}$symmetry where $\hat{R}_6^{(z)}$ is a $2\pi/6$ rotation around the $\hat{z}$ axis and $\hat{\tau}_6$ is a $2\pi/6\Omega$ time translation. The pump field dresses the electronic structure of the molecule, so that its absorption properties change. The complete Hamiltonian of the probed molecule is given by

$$\hat{H} = \hat{H}_0 + \underbrace{\lambda \Re\{z e^{i\omega_p t}\}}_{\hat{W}} \tag{VIII.3}$$

We note that $\hat{H}$ is in fact symmetry broken, because $[\hat{C}_6, \hat{W}] \neq 0$. However, by picking the symmetry breaking degree of freedom, λ, as a synthetic dimension, we are able to recover a symmetry in the symmetry broken system. The Hamiltonian of the system exhibits the symmetry $\hat{C}_6 \cdot \hat{\zeta}_{\omega_p}$ where $\hat{\zeta}_{\omega_p}$ is given by

$$\hat{\zeta}_{\omega_p}(\lambda) = e^{-i\frac{2\pi\omega_p}{6\Omega}} \lambda \tag{VIII.4}$$

Having derived the symmetry of the symmetry broken system, we move on to derive the selection rules. Consider the optical absorption/emission of the system at frequencies $n\Omega + \omega$ where n is an integer. These components of the emission are proportional to the susceptibility of the nth Floquet band $\tilde{\chi}_n(\omega)$ as it was defined previously[4]. To obtain their selection rules, we invoke the invariance of the emission under the real-synthetic symmetry operation:

$$\mathbf{E}(t,\lambda) = \int_{-\infty}^{\infty} d\omega\, e^{i\omega t}\tilde{\mathbf{E}}_{\omega}(\lambda) \tag{VIII.5}$$

$$\hat{C}_6 \cdot \hat{\zeta}_{\omega_p}\tilde{\mathbf{E}}(\lambda,t) = \int_{-\infty}^{\infty} d\omega\, e^{i\omega t} e^{i\frac{2\pi\omega}{6\Omega}} \hat{R}^{(z)}{}_6 \tilde{\mathbf{E}}_{\omega}\left(e^{-i\omega_p \frac{2\pi}{6\Omega}}\lambda\right) \tag{VIII.6}$$

Hence

$$\tilde{\mathbf{E}}_{\omega}(\lambda) = e^{i\frac{2\pi\omega}{6\Omega}} \hat{R}^{(z)}{}_6 \tilde{\mathbf{E}}_{\omega}\left(e^{-i\omega_p \frac{2\pi}{6\Omega}}\lambda\right) \tag{VIII.7}$$

Eq. (VIII.7) is the selection rule imposed by the symmetry $\hat{C}_6 \cdot \hat{\zeta}_{\omega_p}$, in its most general form, i.e., it is valid for any polarization component of the absorbed/emitted field, and it holds for any amplitude of the probe field. We note that it exactly recovers the selection rule that was obtained by perturbation theory. That is, assuming that $\tilde{\mathbf{E}}_{\omega}(\lambda) \propto \lambda$ and $\tilde{\mathbf{E}}_{\omega}(\lambda) \parallel \hat{z}$, simplifies Eq.(VIII.7) to

$$e^{i\frac{2\pi(\omega-\omega_p)}{6\Omega}} = 1 \tag{VIII.8}$$

i.e., the only allowed frequencies are

$$\omega = \omega_p + 6\Omega n \tag{VIII.9}$$

which is exactly equivalent the dark band condition obtained by linear response theory[4]

$$\tilde{\chi}_n(\omega_p) = \begin{cases} 1 & if \quad e^{i\frac{2\pi}{6}n} = 1 \\ 0 & else \end{cases} \tag{VIII.10}$$

However, we emphasize that Eq. (VIII.7) is much more general than previously considered and it applies beyond the regime of linear response.

Supplementary references

1. Neufeld, O., Podolsky, D. & Cohen, O. Floquet group theory and its application to selection rules in harmonic generation. *Nat. Commun.* **10**, 1–9 (2019).

2. Fleischer, A., Kfir, O., Diskin, T., Sidorenko, P. & Cohen, O. Spin angular momentum and tunable polarization in high-harmonic generation. *Nat. Photonics* **8**, 543 (2014).

3. Pisanty, E., Sukiasyan, S. & Ivanov, M. Spin conservation in high-order-harmonic generation using bicircular fields. *Phys. Rev. A* **90**, 43829 (2014).

4. Engelhardt, G. & Cao, J. Dynamical Symmetries and Symmetry-Protected Selection Rules in Periodically Driven Quantum Systems. *Phys. Rev. Lett.* **126**, 90601 (2021).

5. Medišauskas, L., Wragg, J., Van Der Hart, H. & Ivanov, M. Y. Generating Isolated Elliptically Polarized Attosecond Pulses Using Bichromatic Counterrotating Circularly Polarized Laser Fields. *Phys. Rev. Lett.* **115**, 153001 (2015).

6. Neufeld, O., Fleischer, A. & Cohen, O. High-order harmonic generation of pulses with multiple timescales: selection rules, carrier envelope phase and cutoff energy. *Mol. Phys.* **117**, 1956–1963 (2019).

7. Fleck, J. A., Morris, J. R. & Feit, M. D. Time-dependent propagation of high-energy laser beams through the atmosphere. *Appl. Phys.* **10**, 129 (1976).

8. Feit, M. D., Fleck Jr., J. A. & Steiger, A. Solution of the Schrödinger Equation by a Spectral Method. *J. Comput. Phys.* **47**, 412–433 (1982).

9. Hecht, E. *Optics*. (Pearson, 2012).

10. Bordo, E. *et al.* Interlocked attosecond pulse trains in slightly bi-elliptical high harmonic generation. *JPhys Photonics* **2**, (2020).

11. Korneev, P. A. *et al.* Interference carpets in above-threshold ionization: From the coulomb-free to the coulomb-dominated regime. *Phys. Rev. Lett.* **108**, 223601 (2012).

12. Lysne, M., Murakami, Y., Schüler, M. & Werner, P. High-harmonic generation in spin-orbit coupled systems. *Phys. Rev. B* **102**, 081121(R) (2020).

13. Pareek, T. P. Anisotropic spin and charge transport in presence of spin-orbit interaction. *Phys. Rev. B* **66**, 1–4 (2002).

14. Pareek, T. P. & Bruno, P. Magnetic scanning tunneling microscopy with a two-terminal nonmagnetic tip: Quantitative results. *Phys. Rev. B* **63**, 1–5 (2001).

15. Mireles, F. & Kirczenow, G. Ballistic spin-polarized transport and Rashba spin precession in semiconductor nanowires. *Phys. Rev. B* **64**, 24426 (2001).

16. Gu, B. & Franco, I. Optical absorption properties of laser-driven matter. *Phys. Rev. A* **98**, 63412 (2018).
